# Current Options and Future Perspectives for Conversion Coatings on Biodegradable Magnesium Alloys to Control the Biodegradation Rate and Biological Features

**DOI:** 10.3390/biomimetics11040265

**Published:** 2026-04-10

**Authors:** Veronica Manescu (Paltanea), Aurora Antoniac, Julietta V. Rau, Olga N. Plakhotnaia, Marco Fosca, Gheorghe Paltanea, Gabriel Cristescu, Iulian Antoniac

**Affiliations:** 1 Faculty of Material Science and Engineering, National University of Science and Technology Politehnica Bucharest, 313 Splaiul Independentei, District 6, RO-060042 Bucharest, Romania; aurora.antoniac@upb.ro (A.A.); gabriel.cristescu@gmail.com (G.C.); 2 Faculty of Electrical Engineering, National University of Science and Technology Politehnica Bucharest, 313 Splaiul Independentei, District 6, RO-060042 Bucharest, Romania; gheorghe.paltanea@upb.ro; 3 Istituto di Struttura della Materia, Consiglio Nazionale delle Ricerche (ISM-CNR), Via del Fosso del Cavaliere 100, 00133 Rome, Italy; giulietta.rau@ism.cnr.it (J.V.R.); marco.fosca@ism.cnr.it (M.F.); 4 Department of Analytical, Physical and Colloid Chemistry, Institute of Pharmacy, I.M. Sechenov First Moscow State Medical University, Trubetskaya St. 8, Build. 2, 119048 Moscow, Russia; plakhotnaya_o_n@staff.sechenov.ru; 5 Academy of Romanian Scientists, 54 Splaiul Independentei, RO-050094 Bucharest, Romania

**Keywords:** conversion coatings, Mg-based alloys, ceramic coatings, polymeric coatings, bioactive coatings, hybrid coatings, cellular signaling pathways, corrosion behavior, biological response

## Abstract

In the biodegradable metal class, Mg-based alloys are considered the most promising candidates for temporary implant manufacture. However, their high corrosion rate in physiological media is considered a main drawback for clinical translation. Conversion coatings address the limitations of Mg-based alloys and provide a strategy to control corrosion and improve surface biocompatibility. In this review paper, a detailed analysis of various conversion coating techniques, including ceramic conversion coatings based on metals, polymeric conversion coatings, bioactive conversion coatings, and hybrid conversion coatings, is performed. Attention is devoted to the corrosion process and parameters, as well as to the biological response in relation to bioactivity or biocompatibility. The main angiogenic and osteogenic signaling pathways are described based on the analyzed conversion coatings, and the evolution of the cellular response is estimated. Although significant progress has been made in the field, there are still challenges associated with synchronizing Mg alloy degradation with new bone formation and with precisely guiding cell signaling responses to achieve a desired biological response. An overall conclusion of the paper consists of the fact that conversion coatings are an important topic, as they can enhance the surface of Mg-based alloys, making them prone to clinical translation.

## 1. Introduction

Magnesium (Mg)-based alloys exhibit important advantages as biodegradable implant materials due to the fact that they have an elastic modulus with values close to those of the human bone, so no stress shielding effects will occur; a natural biodegradability property, which eliminates the need for a supplementary surgical intervention performed to remove the implant as in the case of permanent implants; and last but not least, the release of bioactive Mg^2+^ ions, which promote tissue regeneration as will be detailed further in this paper. As a critical limitation for Mg-based alloys, it is worth noting their rapid corrosion speed in physiological media, leading to rapid mass loss and potential mechanical integrity issues before the tissue is completely healed or load transfer occurs. Also, as explained later in this review paper, an accelerated Mg alloy degradation profile is accompanied by excessive hydrogen release, leading to gas pocket formation and an alkalinization effect that could negatively impact natural tissue and cell activity. On the other hand, an uncontrolled corrosion process could lead to a non-uniform and localized pitting, which sometimes could even induce implant failure. So, as a direct consequence, it is of utmost importance to correlate the corrosion rate of Mg-based alloys with tissue regeneration.

Conversion coatings are a modern and important research area due to their role in overcoming one of the most significant limitations of Mg-based alloys, as stated before [1,2,3]. These factors negatively affect cell viability and tissue regeneration and, in some cases, can also induce necrosis [4,5]. Depending on the conversion coating, various protective barriers are formed, reducing corrosion rates, stabilizing the interfacial pH, and preventing H_2_ pocket formation [6]. Conversion coatings decrease the corrosion rate of Mg-based alloys by forming a protective interfacial film that modifies the metal–electrolyte interaction. Usually, conversion coatings contain oxides, phosphates, or various insoluble compounds, acting as a physical barrier against aggressive ions such as Cl^−^ and reducing the direct exposure of the Mg-based alloy surface to physiological fluids. Additionally, conversion coatings reduce the anodic dissolution rate by suppressing the localized galvanic effect. On the other hand, the coating porosity, thickness, and microstructure significantly influence its protective efficiency, with implications for Mg alloy corrosion rates. As will be further explored in this paper, conversion coatings could work as functional platforms for incorporating bioactive ions or other chemical species, providing enhanced biological performance and a tunable degradation rate.

The main scope of this paper is to conduct a systematic analysis of conversion coatings for Mg-based alloys, emphasizing key objectives, including the mechanistic aspects of corrosion protection, biological responses, and ion-mediated signaling phenomena. Its main contribution is to identify current limitations and emerging directions, including large-scale preparation, the transition from laboratory analysis to clinical studies, quantitative performance evaluation, selection criteria for conversion coating, and the analysis of hybrid and intelligent coating systems.

The biocompatibility of Mg-based alloys can be enhanced through direct and indirect mechanisms. Regarding the direct process, bioactive conversion coatings based on calcium phosphate could promote apatite formation and trigger specific osteogenic and angiogenic pathways with the ultimate goal of bone defect restoration or blood vessel repair. On the other hand, the indirect process is characterized by a stable microenvironment, supported by the protection provided by the conversion coating, which is favorable to cell adhesion, protein adsorption, and a reduction in inflammatory cytokines [7,8,9].

Last but not least, it can be seen that conversion coatings are of utmost importance in achieving an equilibrium between Mg-based alloy degradation and biological response, making this combination the best candidate for the temporary implant manufacturing domain and one of the most widely used biofunctional materials for clinical translation [10,11,12].

**Figure 1:**
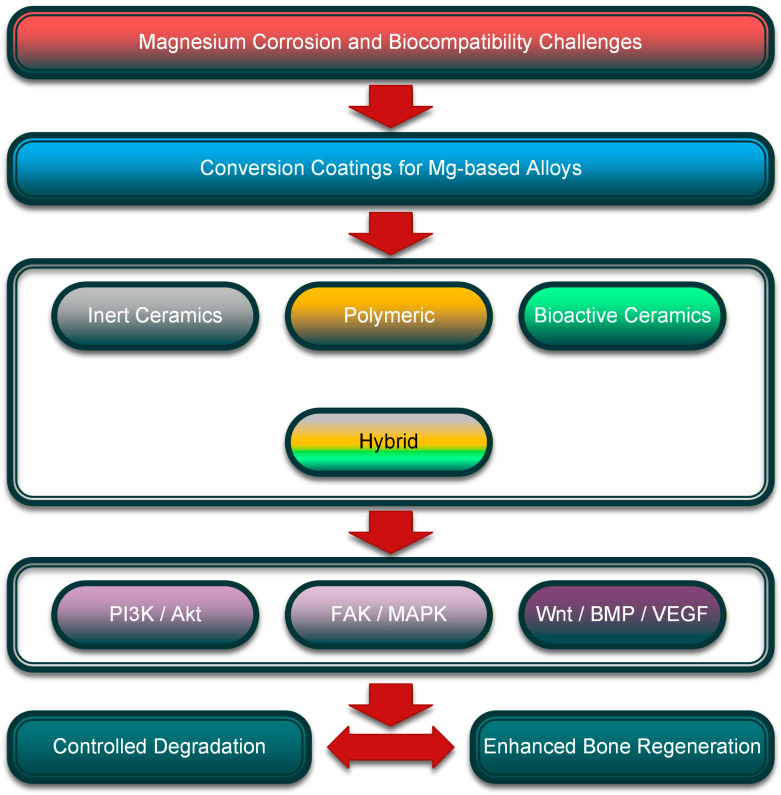
Main research topics developed in the review paper.

In this review paper, a state-of-the-art summary, with particular attention to the general conversion coating techniques suitable for the surface enhancement of Mg-based alloys, will be presented, along with a degradation analysis and biocompatibility assessment. Several types of conversion coatings will be discussed, focusing on their role in improving the alloy’s corrosion resistance and biological features. The main types presented are rare earth or other chemical conversion coatings based on metallic oxides, phosphate conversion coatings, hydrothermal or solvothermal conversion treatments, MAO/PEO with the incorporation of bioactive elements, and some hybrid conversion solutions. Then, an exhaustive classification of coating types (ceramic inert conversion coatings based on metals, polymeric conversion coatings, bioactive ceramic conversion coatings, and hybrid conversion coatings) will be presented, with each method analyzed and examples from the literature provided. In addition, aspects such as industrial preparation and the clinical translation of conversion coatings, the coupling relationship between the mechanical and corrosion properties, and quantitative performance evaluation and selection criteria for conversion coatings will be detailed. A concise evaluation and comparison of the analyzed studies regarding key processing parameters, corrosion resistance, bioactivity/biocompatibility, advantages, and limitations will be conducted and decision indicators will be provided with particular attention to the process parameters and testing conditions for each included study. Last but not least, biological aspects and specific cellular signaling pathways will be analyzed in a separate section, emphasizing key biological triggers, molecular markers, cellular responses, and their main contributions to bone formation. Also, the molecular mechanisms and dose effects of different coating components, such as metallic ions, together with specific signaling pathways, will be detailed. The review paper will end with conclusions, future perspectives, and a key outstanding question box. Figure 1 presents the main topics developed in the review paper.

## 2. State-of-the-Art Summary Regarding Conversion Coating Techniques Used for the Surface Enhancement of Mg-Based Alloys

Conversion coating techniques are based on chemical reactions, ensuring the formation of a protective layer on the surface of Mg-based alloys, thereby increasing corrosion resistance by modulating the alloy’s degradation rate and enhancing biocompatibility [13,14,15,16]. In some cases, there were also improvements in the mechanical properties when the coated alloys were placed in contact with biological fluids [2,17]. In this section, the main conversion coating methods dedicated to Mg-based alloys will be analyzed. Devoted attention will be given to the physical and chemical mechanisms that occur during the coating procedure, and the biological performance in relation to a potential clinical application.

The first conversion coating technology analyzed in the review is the sol–gel method. It is included in the wet-chemical method class and leads to the obtaining of thin layers based on precursor hydrolysis. A clear example comes from Lopez et al. [18] in which a SiO_2_ coating was achieved with the help of tetraethyl orthosilicate hydrolysis, followed by a supplementary curing step performed at a temperature below 200 °C. Castro and Duran [19] reported a very thin layer apparition, with a thickness lower than 5 μm, and underlined the fact that the sol–gel method is a simple and cost-efficient coating route. In addition, this method offers the possibility to add different bioactive dopants such as calcium phosphate, which could improve the overall biocompatibility of the final product as related by Galio et al. [20]. An important key feature of this method is its low-temperature character, which permits the Mg-based alloys to keep their initial bulk properties almost unchanged [2,21]. The disadvantages of the sol–gel method include a long processing time, shrinkage and cracking of the coated film, surface quality, prior preparation, and the method’s sensitivity to environmental humidity.

Another method frequently used in practice is plasma electrolytic oxidation (PEO), a process that occurs under a high-voltage discharge between 200 V and 500 V. It is suitable for obtaining ceramic-based coatings on the Mg alloy surface. Attarzadeh et al. [22] proved that formed oxide layers with a thickness below 100 μm enhanced the corrosion resistance of Mg alloys by leading to a corrosion rate of about 0.6 mm/year in simulated body fluid (SBF). This result is indeed a very good one, since the pure Mg corrosion rate was estimated at about 2.0 mm/year [23]. Drotarova et al. [24] showed that PEO offered the possibility, exactly as in the case of the sol–gel method, to include bioactive elements such as calcium (Ca) or phosphate (PO_4_), a fact that increased the Mg-based osteogenic capabilities. The main drawback of the PEO technique is its need for a high energy source and scalability issues, which underscore its use at the industrial level, as underlined by Hassein Mousavian and Tabaian [25]. Otherwise, it is considered a versatile technology, which permits the fine control of the coating composition [26].

The chemical conversion coating method is based on chemical reactions between Mg-based alloys and a specific solution, resulting in the formation of a protective film. Usually, silicate- and fluoride-based conversion coatings have been reported to be highly efficient at reducing the substrate’s corrosion rate [27]. Double-adherent layers were observed when Mg-based alloys were introduced into hydrofluoric acid (HF) at an optimal concentration [28], while magnesium-phosphate coatings increased the alloy’s bioactivity and corrosion resistance [29]. Hiromoto and Yamazaki [30] showed that a ceramic chemical conversion coating consisting of HAp was generated on the surface of the Mg-Al-Zn (AZ31) alloy and exhibited good cell viability, supporting osteoblast proliferation and adhesion. The main advantages of the chemical conversion coatings consist of the superior adhesion achieved by transforming the metallic surface into a polar and roughened one, the possibility of covering complex geometries, and their cost-effective character. One should also mention some disadvantages, such as the porosity of the coatings; substrate sensitivity connected to the existence of Mg alloys’ second phase, which could be linked to the existence of weak spots in the coating layer; as well as the thickness value, below 5 μm.

The last investigated conversion coating technique is the hydrothermal method, which involves applying high-temperature and high-pressure conditions that enhance crystal growth and chemical reactions at the Mg alloy surface. The hydrothermal method is suitable for producing HAp coatings. Some studies [31,32] showed that, for the Mg-Al-Zn-Mn-Ca alloy (AZ91-3Ca), a reduction in the degradation rate of about 60%, correlated with good biocompatibility, was achieved. Additionally, Wang et al. [33] analyzed the effect of a strontium-doped nanorod/nanowire HAp coating on the Mg-Zn-Zr (ZK60) alloy. An important decrease in corrosion current density was achieved, along with good cell viability, proliferation, and adhesion in simulated oral media. The advantages of the hydrothermal method can be summarized as follows: conversion films are dense and compact, and they exhibit increased adhesion, reduced porosity, and tunable morphology, including nanoflowers, nanorods, or nanosheets. The main disadvantages are the high energy costs associated with high-pressure/high-temperature conditions and the possibility of Mg-based alloy microstructure deterioration due to the increased process temperature.

The field has learned that the chemical conversion method typically involves fluoride/phosphate films that are highly biocompatible but provide reduced long-term corrosion protection. Regarding the sol–gel method, it was noted that bioactive glasses or ceramics are the material of choice when bone growth is the main objective, as they offer enhanced osteointegration. The hydrothermal method is considered the “gold standard” for HAp coatings in the medical field today. Last but not least, the PEO technique should be paired with the use of biocompatible electrolytes that do not produce toxic byproducts. The main challenges that the use of conversion coatings could overcome are the limitation of hydrogen gas emissions, which could delay healing, and the interface instability caused by cells, proteins, or electrolytes present in living bodies, which could easily accelerate Mg alloy corrosion. The main “take-home” criteria are related to the fact that the conversion coating must exhibit a high biocompatibility grade, the surface chemistry has to promote bone cell viability and attachment, the degradation profile must respect the Darcy law for mass transport [10], and ultimately, the sterilization process has to be compatible with the medical use of the implant.

Conversion coatings on Mg-based alloys influence the bioactivity and biocompatibility of the overall implant. For example, some conversion coatings based on Ca-P or other hydrothermal layers generate a bioactive surface that supports the nucleation and growth of HAp after exposure to physiological fluids. In addition, by forming a protective film, the excessive release of Mg^2+^ ions or H_2_ gas is limited. However, coatings obtained from MAO/PEO with bioactive incorporation support cell viability and proliferation very well by providing adequate roughness, porosity, and chemical composition within the biological range for tissue integration. An optimized conversion coating must establish an equilibrium between biofunctional performance and corrosion control, as previously mentioned.

Table 1 presents a synthesis that considers the main coating technologies in relation to Mg-based alloy types, degradation parameters, and biocompatibility.

## 3. Conversion Coating Classification as a Function of Coating Type and Related Aspects

### 3.1. Ceramic Conversion Coatings Based on Metals

Ceramic conversion coatings based on metals are characterized by the fact that, as a consequence of different coating technique applications, metallic ions generate stable oxides or other ceramic compounds at the Mg alloy surface. These films form protective layers, which provide increased corrosion resistance and good osteoconductive properties.

One of the most commonly used conversion coatings in practice is that based on rare earths (RE) such as yttrium (Y), cerium (Ce), and neodymium (Nd) [34,35]. Regarding cerium dioxide (CeO_2_), it is worth noting that it exhibits significant antioxidant properties and can reduce reactive oxygen species (ROS), thereby positively impacting osteogenesis and inflammatory processes. Usually, REs’ ions chemically react with hydroxide ions OH^−^, which is a direct result of Mg-based alloy corrosion processes, and generate a protective film consisting of metal hydroxide at the substrate surface. Regarding the Nd-based conversion coatings, an electrochemical process is involved, and Nd^3+^ ions are directly guided to the Mg alloy surface. It results in an Nd_2_O_3_ oxide layer that provides enhanced corrosion protection and a passive, stable surface. In this way, the initial burst of hydrogen (H_2_) release is minimized, and pH spikes are limited. Rudd et al. [36] investigated the effect of a conversion coating based on Ce oxide and hydroxide formed on the surface of Mg-Y-Nd-Zr (WE43). The authors found an increase in corrosion resistance based on cathodic site sealing in conjunction with reduced electrochemical activity. Another study performed by Dabala et al. [37] sustained their findings based on a similar Ce-based conversion coating with a mixed composition consisting of Ce oxide and hydroxide formed on the surface of the Mg-Al-Zn (AZ63) alloy. In addition, Rocca et al. [38] analyzed two chemical conversion methods based on Ce and phosphate–permanganate treatment formed on the surface of Mg-Al alloys. Their main conclusion was that these protective layers were highly effective at delaying the onset of corrosion. Han et al. [39] made an Y-based conversion coating on the Mg-Al-Zn (AZ91D) alloy in combination with a phosphating process. In this way, a dense and stable Y-P conversion coating occurs. It was concluded that it offers high corrosion resistance. Cui et al. [40] applied a composite conversion coating based on carboxylic acid-neodymium on the surface of the Mg-Al-Zn alloy, followed by a hydrothermal treatment to stabilize the protective film. In ref. [41], a modified version of this method is presented, consisting of developing a Nd-based oxide/hydroxide layer on the surface of the same Mg alloys as in ref. [40], with an important effect on diminishing the entire corrosion behavior of the surface.

Plasma electrolytic oxidation could be used in combination with a metal-doping procedure. This complex procedure is possible when the electrolytes are introduced to metals such as Titanium (Ti) or Zirconium (Zr). As a direct consequence, the PEO coatings contain mandatory TiO_2_ or ZrO_2_ oxide as a function of the metal doping type. The mechanism of this process is based on the incorporation of the metallic ions into a molten ceramic matrix, leading to the formation of a composite layer. In this direction, Penuela-Cruz et al. [42] made a composite PEO coating containing MgO and TiO_2_ oxides on a bare Mg alloy. It was also evidenced that this oxide-rich surface film has a beneficial effect on the reduction in the corrosion rate of magnesium alloys. White et al. [43] deposited a crystalline TiO_2_ layer on the surface of the Mg-Al-Zn (AZ31) alloy using the PEO method. The authors demonstrated that the protective conversion coating significantly reduced the corrosion rate. Halimovic et al. [44] prepared a TiO_2_-doped PEO-obtained coating sealed with polylactic acid (PLA) on the Mg-Al-Zn (AZ31) alloy. The protective character against the Mg degradation was assumed to be a consequence of the increased wear resistance of the TiO_2_ oxide with the hydrophobic, pore-filling characteristics of PLA. It was concluded that the combination of ceramic and polymeric mixed coating is a promising strategy for biocompatible implant manufacture. Wang et al. [45] prepared a ZrO_2_-incorporated PEO coating on the Mg-Li (LA103Z) surface. A stress-induced phase transformation, which decreased the corrosion speed and maintained the structural integrity of the alloy, was attributed to the ZrO_2_ doping procedure. Li et al. [46] manufactured ZrO/MgO composite coatings based on PEO technology and showed that by changing the pH, a transition between interconnected and isolated pore structures could be achieved. In addition, a reduced value of the current density was obtained.

A special conversion coating, which promotes biomolecule adhesion, consists of applying potassium stannate to generate tin oxide layers on the Mg surface. In this way, the initial burst of the pH is reduced, and the alkalinization of the environment is maintained within the biological limits, facilitating the adhesion of biomolecules. Based on this method, Lin et al. [47] obtained a crystalline MgSn(OH)_6_ protective layer on the Mg-Al-Zn (AZ61) alloy. A suppression effect on the anodic dissolution of the magnesium matrix was observed, along with a corrosion process dependent solely on nucleation density and grain morphology. It was concluded that this chemical conversion treatment is efficient by considering the corrosion effect, but further research is necessary to validate the coating’s biological application. The self-healing property of a stannate-based coating developed on the Mg-Al-Zn-Mn (AZ91D) surface was shown by Hamdy and Butt [48]. This effect consisted of the elimination of mechanical defects or scratches as a consequence of the surface passivation, leading to the recovery of the surface’s initial quality. In addition, an increased corrosion resistance was estimated together with an adequate presumed biocompatibility that will recommend the coatings as adequate in the cardiovascular and orthopedic domains. Kumar et al. [49] prepared a double coating system consisting of Ni-P layer deposition over a stannate-based conversion coating on the Mg-Al-Zn-Mn (AZ91D) surface. It was concluded that the first conversion coating was beneficial for the second procedure, leading to a uniform and defect-free Ni-P deposition process, offering an increased wear resistance and a decrease in the corrosion rate simultaneously.

Conversion coatings based on metals could also be achieved based on a hybrid sol–gel method combined with metallic nanoparticle reinforcement. Usually, an inorganic sol–gel network is reinforced with metal oxide nanoparticles such as MgO or ZnO with bioactive or antibacterial properties. Talha et al. [50] used a self-assembled silane matrix reinforced with ZnO nanoparticles as a conversion coating for biomedical-grade magnesium alloy Mg-Al-Zn (AZ31). The authors demonstrated that the combined effect of the silane network and the nano-filler bridged interfacial defects, generating a more compact and hydrophobic barrier that inhibits the anodic dissolution of the Mg substrate. Rodriguez-Alonso et al. [51] developed a hybrid sol–gel coating treatment combined with the doping of biocompatible corrosion inhibitors for Mg-Al-Zn (AZ61) alloys. This combined approach merged the structural barrier protection with active inhibition, leading to a significant advancement in generating biomimetic surface modifications for orthopedic applications.

**Figure 2:**
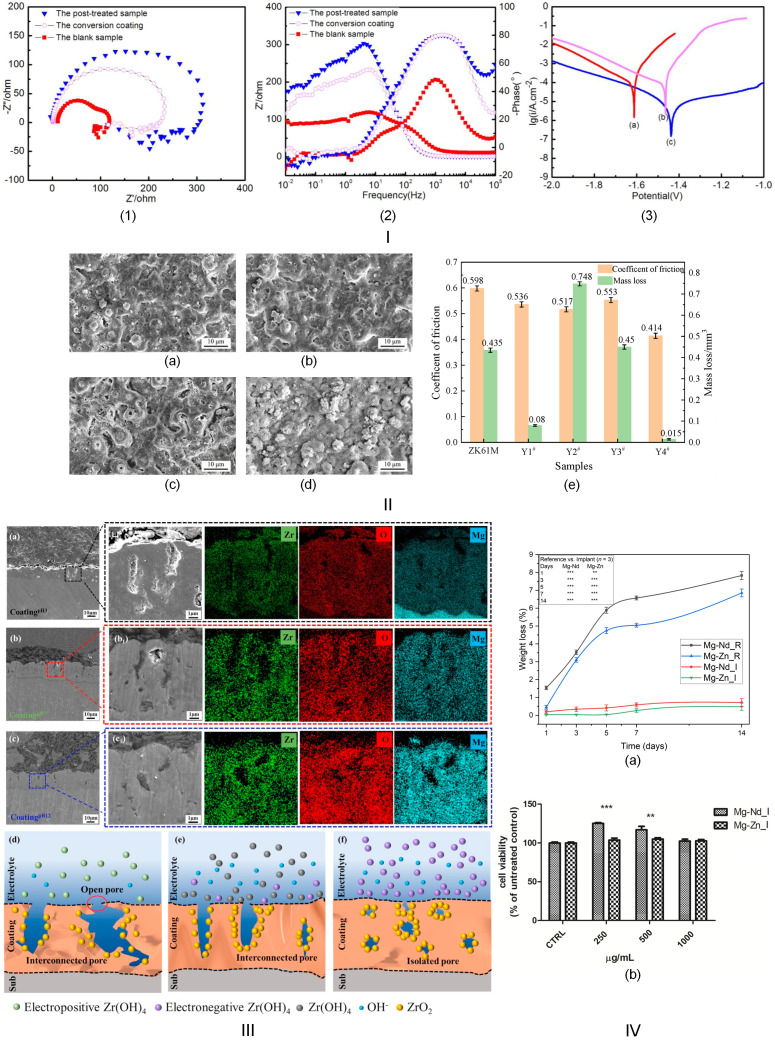
Degradation and biological aspects related to Mg-based alloys with ceramic conversion coating based on metals. (**I**) Electrochemical corrosion analysis on Mg-Al-Zn-Mn (AZ91D) Y- and YPO4-based coatings: (**1**) Nyquist plots; (**2**) Bode phase plots—23C, 3.5 wt.% NaCl solution; (**3**) Tafel variations [(**a**) uncoated alloy; (**b**) Y-coated alloy (10g/L Y(NO_3_)_3_ at 30 °C for 50 min); (**c**) YPO_4_—coated alloy (10g/L Y(NO_3_)_3_ at 30 °C for 50 min and then in solution containing 1.5% mass fraction of NH_4_H_2_PO_4_ at 80 °C for 120 s] [39]. (**II**) Mg-Zn-Zr MAO coated with different concentrations of Y(NO_3_)_3_: scanning electron microscopy images (**a**) 0 g/L, (**b**) 0.15 g/L, (**c**) 0.45 g/L, (**d**) 0.75 g/L; (**e**) friction coefficient and mass loss of MAO coating Y1^#^ (0 g/L), Y2^#^ (0.15 g/L), Y3^#^ (0.45 g/L), and Y4^#^ (0.75 g/L) [34]. (**III**) Cross-sectional images of different coatings (ZrO_2_/MgO) on Mg-Al-Zn-Mn (AZ91D) and associated EDS map as a function of electrolyte pH: (**a**,**a1**) coating pH = 3, (**b**,**b1**) coating pH = 7, (**c**,**c1**) coating pH = 1 2; schematic structure diagram of pore growth in different coatings: (**d**) coating pH = 3, (**e**) coating pH = 7, and (**f**) coating pH = 12 [46]. (**IV**) Mg-Nd and Mg-Zn alloys uncoated and coated with MgF_2_: (**a**) weight loss after 1, 3, 5, 7, and 14 days of immersion in SBF (** *p* < 0.01; *** *p* < 0.001); (**b**) viability of osteoblast cells after 24 h interaction with MgF_2_ coated alloys (** *p* < 0.01, *** *p* < 0.001) [28]. All the images contained in this figure are under CC BY 4.0 or CC BY-NC-ND 4.0 copyright license.

An effortless conversion method for coatings based on metals consists of the formation of a magnesium fluoride (MgF_2_) coating, which is usually used in biomedicine. It is based on Mg alloy immersion in hydrofluoric acid (HF) with a given concentration, which can tune the ceramic conversion coating thickness and properties. The MgF_2_ protective film is chemically stable and acts as an electrical insulator, which blocks the movement of corrosive electrolytes. Manescu (Paltanea) et al. [28] analyzed the biofunctionality of two Mg-based implants (Mg-Nd and Mg-Zn), which exhibited a conversion coating of MgF_2_ as an indirect result of a new manufacturing route. It was shown that this conversion coating offers a reduced mass loss when the samples were immersed in SBF as well as an increased biocompatibility demonstrated by in vitro analysis performed on human-patella-derived osteoblasts. In the study of Quan et al. [52], the effect of MgF_2_ conversion coating was investigated in the case of the Mg-Nd-Y-Zn-Zr alloy. The authors proved that the MgF_2_ conversion layer serves as a strong physical barrier that significantly slows the anodic dissolution and ion release from the rare earth containing magnesium substrate. Figure 2 presents degradation and biological results for Mg-based alloys exhibiting a ceramic conversion coating based on metals.

It can be noticed that ceramic conversion coatings based on metals must exhibit biomimetic and bioactive behavior to enhance the biocompatibility of Mg-based alloys and reduce their corrosion rate. One can immediately observe that incorporating metal oxides (ZnO, CeO_2_, and Nd_2_O_3_) or metallic nanoparticles (Ti and Zr) resulted in a much thicker, uniform, pore-free layer. In the case of doping with corrosion inhibitors such as REs, self-healing can be achieved, eliminating microanodic sites. The field has also learned that an efficient conversion coating for biomedical applications should exhibit electrochemical stability, a low dissolution rate, and an adequate biological response that promotes cell adhesion and proliferation.

The current challenges arise from the fact that, in almost all cases, a mismatch between the Mg thermal expansion coefficient and the ceramic coating’s rigidity can occur. This fact is directly linked to the formation of microcracks or surface delamination. Another important challenge is associated with the synchronization between Mg-based alloy biodegradability and new bone formation, as otherwise foreign body responses may occur. Last but not least, it is worth mentioning that complex processes such as PEO, the hybrid sol–gel method, and nanoparticle reinforcement are characterized by the good quality of the protective layers but could hardly be applied in the case of complex geometries in orthopedic implants.

The most important “take-home” criteria are: a reduced porosity of the coatings must be combined with a high density to isolate the Mg-based substrate efficiently; the conversion coating must be self-healing, a fact that is identified in a rapid recovery of the corrosion current density; an improved interfacial adhesion energy should be maintained to prevent the delamination process; and conversion coatings based on metals should be highly biocompatible.

### 3.2. Polymeric Conversion Coatings

Polymer-based conversion coatings are considered today to be a modern approach to modifying the Mg alloy surface [53,54]. This process involves a chemical transformation in which the polymer’s functional groups form a covalent bond with the metal oxide layer during a normal passivation process.

One of the methods used in practice is the in situ oxidative polymerization procedure, which is based on the intrinsic electrochemical activity of the Mg to start and continue a polymerization reaction that occurs directly at the metal–solution boundary, resulting in a chemically stable and adherent protective film formation. The main mechanism of this method is the anodic dissolution of Mg, which is coupled with cathodic chemical reactions such as water reduction, leading to hydroxyl ion generation. As a final result, an alkalinization of the medium placed in the surface vicinity is achieved. This process initiates an oxidative precipitation of certain monomers, leading to the nucleation and growth of polymer chains such as polydopamine (PDA), polypyrrole (PPy), and polyaniline (PANI) directly on the Mg substrate. In this way, it results in a polymeric coating linked to the Mg(OH)_2_/MgO conversion layer. Meng et al. [55] used the chemical conversion/self-polymerization method to develop a Zn^2+^-loaded PDA coating on the surface of Mg-Al-Zn (AZ31B). Regarding the alloy degradation profile, it was observed that the PDA coating acts as a barrier, improving corrosion resistance, while the Zn^2+^ ions generated by the PDA coatings led to grain refinement, further increasing corrosion resistance. The alloy’s biocompatibility was tested based on endothelial cells. The authors found that the Zn^2+^-loaded PDA coating was beneficial to cell adhesion and proliferation, demonstrating an increased biocompatibility compared to the uncoated Mg-Al-Zn alloy. Liu et al. [56] developed a method based on a double treatment in two steps: first, a hydrofluoric acid immersion was performed, followed by a self-polymerization process involving PDA. The MgF_2_/PDA reduced the corrosion rate and hydrogen evolution by a greater degree than the MgF_2_-coated Mg-Zn-Y-Nd alloy. An improved biological response was observed with the hybrid coating in in vitro tests on endothelial cells (EA.hy926) and vascular smooth muscle cell lines (A7r5). It was concluded that a superior hemocompatibility was possible with the MgF_2_/PDA-coated alloys, a fact that was directly linked to a rapid endothelialization process. A reduction in the risk of thrombosis was foreseen for this type of conversion coating, making it suitable for use in cardiovascular surgery. The advantages of this method include its simplicity and the fact that it does not require expensive equipment. In addition, this chemical conversion coating procedure could also be applied in the case of complex geometries. As a drawback, it is worth noting that this method depends on oxygen concentration, pH, and alloy composition, as all these factors influence the kinetics of polymeric chain growth concomitantly with Mg dissolution. In situ polymerization offers a robust and stable route for generating polymer-based conversion coatings on Mg-based alloy surfaces.

Chemically induced polymer–metal complex conversion layers are characterized by the following coupled processes: interfacial precipitation, substrate dissolution, and ionic interactions. In this manner, a hybrid organic–inorganic interfacial structure could be achieved. The main mechanism involved in this chemical process is Mg anodic dissolution in conjunction with the cathodic reactions, as in the in situ oxidative polymerization method. The most commonly used polymers in correlation with this chemical conversion strategy are poly(acrylic acid), alginate, and chitosan. Song et al. [57] made a polymeric coating based on a chemical conversion method coupled with microcapsule incorporation on the Mg-Al-Zn (AZ91D) alloy. These microcapsules were loaded with benzotriazole, which is a well known corrosion inhibitor. It was concluded that the developed coating exhibited self-healing behavior, involving microcapsule rupture and inhibitor release, with a significant effect on corrosion suppression. Chemically induced polymer–metal conversion layers exhibit multiple corrosion-protective mechanisms. For instance, the inorganic component is mainly a barrier against corrosive media, while the polymeric network acts as a reduction agent that decreases the permeability to aggressive species such as chloride ions (Cl^−^) and can maintain water in a bound state with a beneficial effect on localized corrosion processes. The most important advantage of this method is characterized by the absence of high-temperature processing or external electric input. A careful optimization of the solution chemical elements and behavior must be conducted to achieve a uniform and high-quality coating without major defects. In conclusion, this method offers a complex and versatile strategy for reducing the corrosion speed of Mg-based alloys.

Sol–gel-derived organic–inorganic conversion coatings for Mg-based alloys are characterized by the formation of hybrid networks via hydrolysis and condensation reactions between metal and organic precursors. This process starts with a sol preparation that contains hydrolysable precursors such as silanes. After hydrolysis, silanes generate reactive silanol (Si-OH) groups that undergo condensation reactions, forming a three-dimensional Si-O-Si network. When this process is applied to Mg alloys, MgO/Mg(OH)_2_ provides reactive sites for interfacial bonding. In addition, the formation of Mg-O-Si bonds arises from condensation between hydroxylated Mg surface species and silanol groups. Nezamdoust et al. [58] prepared a silane-based sol–gel (tetraethoxysilane/3-glycidoxypropyltrimethoxysilane) reinforced with a nanoceramic particles (TiO_2_ and ZrO_2_) coating on the surface of Mg-Al-Mn (AM60B). It was observed that incorporating inorganic metallic oxide nanoparticles increased the corrosion resistance of the silane matrix and provided, in addition, good electrochemical stability in a 0.05 M NaCl solution. It was concluded that optimizing nanoparticle loading could yield coatings with tunable corrosion protection against Cl^−^ ions. The main advantage of this method is its tunable nature as a function of precursor chemistry, pH, curing conditions, and water-to-alkoxide ratio. In this way, the coating film thickness, porosity, and crosslinking density could be easily controlled. In addition, bioactive molecules, nanoparticles, or corrosion inhibitors could be incorporated into the sol. Thus, as a direct consequence, complex coatings could be achieved. The main disadvantage of this method is the need to obtain a coating free of defects, and sometimes pre-treatments are required.

Electrochemically assisted conversion with polymer formation is achieved by applying an external electrical field. In this way, both in situ polymer synthesis and Mg alloy substrate modification are controlled, resulting in a hybrid organic–inorganic coating chemically bonded to the Mg substrate. Usually, the procedure is conducted in an electrochemical cell with Mg alloy as the cathode and integrates electrochemical classical conversion phenomena with polymerization reactions. Under cathodic polarization conditions, a water reduction process occurs at the Mg alloy surface. Hydroxyl ions and an increase in pH are noticed in the vicinity, and Mg(OH)_2_ conversion layer formation is enhanced. Concomitantly, an anodic polymerization occurs as a function of the monomers present in the electrolyte solution. The Mg^2+^ ions’ emission is combined with polymer formation, facilitating their incorporation into the newly formed polymeric matrix. This method is suitable for conductive polymers such as PANI and PPy. Huang et al. [59] used the electrochemically assisted conversion method to obtain a dense PPy coating on the surface of the Mg-Al-Zn (AZ31) alloy. It was observed that the compact organic film significantly reduced the anodic dissolution of the Mg substrate. The deposition parameters were carefully checked to obtain a high-quality and pore-free coating layer. A much lower corrosion current density was reported for the coated samples compared to the uncoated samples. Peng et al. [60] applied one-step cyclic voltammetry dedicated to the electropolymerization of a PPy/silane composite film to modify the surface of the Mg-Al-Zn (AZ31) alloy. A synergistic effect, in which silane was used to obtain sealing and improved adhesion, and PPy behaved as a protective layer, was evidenced. An increase in the pore and transfer charge resistances from 2.59 × 10^3^ Ω·cm^−2^ to 2.46 × 10^6^ Ω·cm^−2^ and 9.70 × 10^3^ Ω·cm^−2^ to 2.87 × 10^6^ Ω·cm^−2^ respectively when the volume fraction of silane coupling agent was 15%, simultaneously with a reduced value of the corrosion current density from 1.833 × 10^−7^ A cm^−2^ to 5.935 × 10^−10^ A cm^−2^ with the best performance for 15% silane agent, were achieved in comparison with the Mg alloy samples coated with simple PPy. The main advantage of this method lies in the fact that a dense and adherent layer is formed, offering increased corrosion protection with the polymer also acting as a corrosion inhibitor. By controlling the deposition parameters, it is possible to achieve a uniform coating even for complex shapes or intricate geometries. However, this technique has some challenges. The first one is associated with the possibility of excessive anodic dissolution, which is directly linked to high H_2_ emissions that could induce pores or defects in the protective films. Careful control of the electric potential, electrolyte composition, and treatment duration is essential for producing a high-quality conversion coating. To summarize the main findings regarding the electrochemically assisted method with polymer formation, it can be noted that this procedure is controllable and versatile, yielding adherent, multifunctional coatings.

Self-assembled reactive polymer conversion layers (LbL) are a special surface modification in which polymers spontaneously organize and chemically react with the Mg alloy surface to form a strong bond conversion coating. The formation mechanism is based on other cases of Mg dissolution and surface hydroxylation, leading to the well known Mg(OH)_2_/MgO layer. In addition, the hydroxylated material interface exhibits many reactive sites that permit interaction with certain functional groups, such as silanol, carboxyl, catechol, or phosphonate groups, present in polymers. Finally, linkages between the Mg substrate and the above-mentioned groups generate Mg-O-C, Mg-O-Si, and Mg-O-P linkages. This process involves the formation of a self-assembled film chemically bound to the substrate. Liu et al. [61] prepared a multilayer composite coating consisting of silane/graphene oxide (GO) composite coating on the surface of the Mg-Al-Zn (AZ91) medical-grade alloy using a layer-by-layer self-assembly method. To investigate the coating’s corrosion protection, the Mg^2+^ ion concentration was measured in SBF solution. It was found that the coated samples offered very low values of 0.12 mM/cm^2^ (24 h) and 0.34 mM/cm^2^ (72 h), respectively. In addition, reduced H_2_ release was observed in the study for coated samples. The in vitro cytocompatibility of the developed coating was analyzed using the MG-63 human bone osteosarcoma cell line. It was concluded that the cell viabilities of the 50% and 100% extracts were higher than 75%. This fact was considered a standard of good cytocompatibility for coated alloys. Kunjukunju et al. [62] applied an alkali/fluoride surface treatment followed by a layer-by-layer self-assembly procedure. It resulted in a polyelectrolyte multilayer assembly of poly(allylamine hydrochloride) (PAH) and poly(lactic-co-glycolic acid) (PLGA) on the surface of the Mg-Al-Zn (AZ31) alloy. The EIS analysis revealed values for the coating resistance of 23,613 Ω, polymer resistance of 80,666 Ω, and substrate resistance of 1951 Ω. The corrosion current density varied between 7.97 × 10^−6^ A/cm^2^ and 3.09 × 10^−7^ A/cm^2^, indicating a much nobler character for the coated samples. Human mesenchymal stem cells (hMSCs) exhibited increased adhesion and proliferation for the coated samples compared to the uncoated alloy. The main advantage of this method is that it provides strong interfacial bonding between the coating and the substrate via a very simple procedure that requires only immersion in a given solution under mild conditions. On the other hand, this modern process depends on oxidation conditions, pH, polymer concentration, and dissolved oxygen. All these factors have a great impact on interfacial reactions and assembly kinetics. In conclusion, by combining spontaneous molecular organization with interfacial bonding, thin, adherent, and biofunctional coatings can be integrated into the substrate.

**Figure 3:**
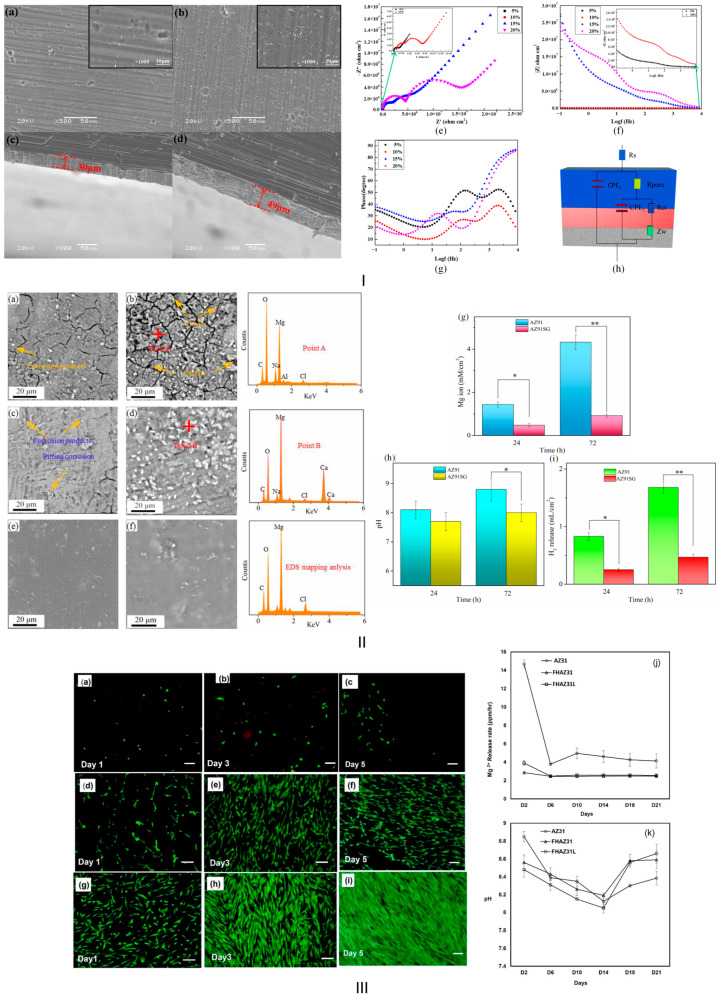
Degradation and biological investigations for polymeric conversion coatings on Mg-based alloy surface. (**I**) SEM images of surface and cross-sectional structure and EIS test related to PPy/silane coating obtained through cyclic voltammetry on Mg-Al-Zn (AZ31) alloy: surface morphology (**a**) in the absence or (**b**) in the presence of an electric field, cross-sectional image (**c**) in the absence or (**d**) in the presence of an electric field; EIS analysis as a function of different silane coupling agents in 3.5 wt.% NaCl: (**e**) Nyquist diagram, (**f**,**g**) Bode diagram, (**h**) equivalent electric circuit [60]. (**II**) SEM images coupled with EDS analysis and Mg^2+^ ion release measurements performed on Mg-Al-Zn (AZ91) alloy using layer-by-layer self-assembly coating based on silane and GO: SEM + EDS—uncoated alloy/silane-coated/silane-GO coated (**a**,**c**,**e**) immersion 24 h; (**b**,**d**,**f**) immersion 72 h; (**g**) Mg^2+^ ion release; (**h**) pH variations; (**i**) H_2_ emission evolution (* *p* < 0.05, ** *p* < 0.01) [61]. (**III**) Biological microscopy images and cumulative amount of Mg^2+^ released from LbL coating on Mg-Al-Zn (AZ31) alloy: live/dead cells taken at 1, 3, and 5 days for (**a**–**c**) uncoated Mg alloy, (**d**–**f**) MgF_2_/Mg(OH)_2_ coated, (**g**–**i**) MgF_2_/Mg(OH)_2_+ PLGA/PAH LbL coated (scale bar: 200 μm); in vitro biodegradation tests over 21 days: (**j**) Mg^2+^ release, (**k**) pH profile variation [62]. All the images contained in this figure are under CC BY 4.0 or CC BY-NC-ND 4.0 copyright license.

Regarding polymeric conversion coatings, the field has learned that conducting polymers such as PPy or PANI act as electronic buffers, maintaining a high electrochemical potential. This fact protects Mg alloys even with a low-quality conversion coating. The most stable conversion coatings are considered those in which Mg^2+^ ions suffer a crosslinking with natural polymers or the Mg surface initiates the polymerization of conducting polymers. Additionally, it is well accepted that silanes offer the best coating adhesion; the inclusion of ceramic nanoparticles generates a tortuosity-like structure, and, last but not least, the introduction of microcapsules provides a self-healing surface property. Critical challenges arise with H_2_ emissions from coatings that block water, as gas accumulation can increase the pressure between the protective layer and the substrate, potentially damaging the coating. Another problem is that organic polymers bind to MgO/Mg(OH)_2_ sites in SBF, but in water, things will look different, since water molecules bind to the same sites. Sometimes delamination could occur. Last but not least, the complex layer-by-layer method or sol–gel-based strategies are difficult to apply to implants with complex geometries. A successful coating is defined by the covalent anchoring—bonding energy value; dynamic response—active recovery; gas diffusivity—diffusion coefficient; and biological signaling—cell adhesion rate. These parameters are among the most important “take-home” criteria to consider when developing a high-quality, biocompatible polymeric conversion coating. In Figure 3, experimental findings regarding polymeric conversion coatings on Mg-based alloy surfaces are detailed.

### 3.3. Bioactive Ceramic Conversion Coatings

The conversion coatings used to obtain bioactive ceramic conversion layers are based on chemical or electrochemical transformations that occur at the surface of Mg-based alloys, simultaneously with in situ precipitation of bioactive phases such as hydroxyapatite (HAp), beta-tricalcium phosphate (beta-TCP), and bioglasses (BGs) [63,64].

Chemical conversion in SBF or supersaturated ionic solutions is based on the Mg alloy’s reactivity in aqueous media and the precipitation of the calcium phosphate (CaP) phase at the metal–solution interface, generating a bioactive conversion coating. As in the other conversion methods, Mg undergoes anodic dissolution, while at the cathode, water dissolution produces hydroxyl ions. A direct result is the localized increase in pH at the interface. Medium alkalinization enhances the nucleation of the CaP compound. As pH increases, the solution becomes saturated with biocompatible ions such as Ca^2+^ and PO_4_^3-^ in SBF. The supersaturation phenomenon is linked to the apparition of amorphous calcium phosphate, which is transformed into a much more thermodynamically stable phase called hydroxyapatite (HAp). In some cases, Mg^2+^ ions are incorporated into the calcium phosphate lattice, and the so-called “Mg-substituted apatite” is formed. Prabhu et al. [65] prepared a chemical conversion coating based on CaP through immersion in SBF. It resulted in a ceramic dicalcium phosphate dihydrate (DCPD/brushite) protective layer chemically bonded to the surface of the Mg-4Zn alloy. A significantly nobler character was noticed for the coated samples (i_corr_: 1.65 × 10^−3^ mA, E_corr_: −0.41 V) compared to the bare Mg-4Zn alloy (i_corr_: 76.12 × 10^−3^ mA, E_corr_: −1.51 V). The investigation demonstrated excellent bio-mineralization potential, resulting in a bone-like apatite layer on the Mg alloy coating after SBF immersion. The main advantage of chemical conversion in SBF or other supersaturated solutions is that it yields biomimetic, substrate-driven calcium phosphate ceramics that provide corrosion protection and high bioactivity, facilitating new bone formation. The main challenges associated with this method are related to the fact that the method is sensitive to environmental parameters, temperature, pH, and ion concentration. In addition, uncontrolled Mg corrosion is linked to excessive hydrogen emissions that can induce unwanted porosity. In summary, this method enables the in situ formation of bioactive, chemically bonded layers with high potential for biomedical applications.

Phosphate conversion coatings using CaP chemistry are among the most widely used techniques worldwide and form a bioactive ceramic layer on the surface of Mg-based alloys. Usually, the Mg alloy is immersed in an aqueous solution containing calcium ions (Ca^2+^) and phosphate ions (PO_4_^3-^). This solution is supplemented with Ca^2+^ ions and buffering elements to control the overall pH. As in other cases, the Mg undergoes classical anodic dissolution concomitantly with the generation of hydroxyl ions at the cathode. The medium alkalinization triggers the CaP compound precipitation as follows: firstly, dicalcium phosphate dihydrate (CaHPO_4_·2H_2_O, DCPD) or amorphous CaP nucleate, then, as a result of a continuous ion exchange, these initial phases transform into more stable and less soluble types such as octacalcium phosphate (OCP) or HAp. Zaludin et al. [66] prepared a simple bioactive conversion coating on pure Mg (99.9% purity) (UC) through immersion in a phosphating solution in two steps. Firstly, a ceramic dicalcium phosphate dihydrate coating (PRI) was obtained, and secondly, a HAp layer was prepared (SEC) based on an alkaline treatment. Taking into consideration the electrochemical corrosion analysis, the coated samples were characterized by a corrosion current density of 25.25 μa/cm^2^ and an electric corrosion potential of −1.62 V compared to the bare Mg alloy (i_corr_ = 185.76 μA/cm^2^, E_corr_ = −1.84 V), clearly showing a much nobler character with an increased corrosion resistance for the coated samples. In addition, it was established that the CaP coating had a porosity of 0.3%, making it a very good candidate for the bioactive coating domain in orthopedic implants. It was concluded that the processing parameters are of utmost importance in obtaining a high-quality coating. The main advantage of this method is its dual character, based on bioactive behavior and concomitant increased corrosion protection, as stated in ref. [66]. However, careful control of the conversion parameters is necessary to optimize coating growth while maintaining substrate stability and preventing the formation of non-uniform or poorly adherent coatings.

Hydrothermal and solvothermal conversion treatments for Mg-based alloy surfaces are characterized by forming a high-quality CaP coating at elevated temperatures between 80 °C and 200 °C under appropriate pressure conditions. The autogenous pressure sustains rapid ionic diffusion and an accelerated interfacial reaction. Regarding solvothermal treatments, mixed or aqueous solvents are used, which facilitate greater control over reaction pathways and solubility. In addition to the anodic dissolution of Mg and the generation of OH^−^ ions, under hydrothermal conditions, a highly conducive medium with increased pH is created. This leads to the precipitation of CaP phases. In addition, working at high temperatures sustains the ceramic crystal growth and nucleation, leading to crystallized phases such as β-TCP, HAp, or mixed Ca-Mg phosphates [67]. Dong et al. [68] manufactured a CaP-based chemical conversion coating via hydrothermal treatment at different temperatures on the Mg-Y-Nd-Zr (WE43) alloy. It was found that the ceramic DCPD coating at 150 °C exhibited a dense, crack-free structure. Additionally, the hydrogen evolution rate of about 7.9 × 10^−4^ mL·cm^−2^·h^−1^, the corrosion rate of 0.07 mm/year, and the corrosion current density of 2.97 μA·cm^−2^ were experimentally determined for the 150 °C hydrothermal-treated samples. It was concluded that i_corr_ decreased by 92.8%, and H_2_ emission was one order of magnitude lower than in the case of the uncoated samples, making this type of conversion coating suitable for biological use. The principal advantages of this conversion coating method are highlighted by the high crystallinity and low porosity of the bioactive film it produces, offering increased corrosion protection and high osteoconductivity. As in the other procedure cases, the method is strongly dependent on the control of process parameters, because at high temperatures, Mg dissolution is enhanced and H_2_ emission could increase, impacting the quality of the formed layer. It can be concluded that the hydrothermal or solvothermal route enables the formation of crystalline, strongly adherent ceramic coatings on the Mg alloy surface.

Electrochemical conversion based on an electrodeposition-assisted CaP layer depends on the application of an electrical potential, which controls the interfacial chemical reactions well and promotes the in situ precipitation of CaP directly on the Mg-based alloy surface. In this method, Mg serves as the cathode, inserted into an electrolyte containing Ca^2+^ and PO_4_^3−^. A water reduction process occurs at the electrode surface, leading to a pH increase directly in the vicinity of the Mg substrate. The saturation of the electrolyte with CaP compounds is achievable in highly alkaline media, initiating the growth and nucleation of a chemically integrated conversion layer, with mixed Mg-Ca phosphate phases due to classical Mg substrate dissolution and surface hydroxylation. Han et al. [69] investigated the importance of a duplex CaP conversion coating, prepared by an electrochemical procedure followed by a chemical conversion treatment on the pure Mg surface. The ceramic conversion coating consisted of a MgO inner layer combined with a CaP conversion top coat. The potentiodynamic polarization curves offered a comparison between MgO-coated and MgO-CaP-coated samples. In the case of MgO-coated probes, an E_corr_ of −1.77 V and i_corr_ of 1.9 × 10^−5^ A/cm^2^ were obtained, while for the duplex conversion coated samples, an improved corrosion behavior was noticed, sustained also by E_corr_ of −1.55 V and i_corr_ of 1.62 × 10^−7^ A/cm^2^ values. It was concluded that the MgO-CaP conversion coating closed the existing micropores and cracks in the film, thereby enhancing corrosion resistance and reducing H_2_ emission. This procedure offers the advantage of obtaining a uniform coating growth, suitable also in the case of complex geometries. A balance between electrochemical parameter values is essential to achieve adherent, dense, and phase-controlled coatings, and attention should be paid to limiting H_2_ emission to prevent coating imperfections. Combining substrate-driven chemical reactions with electrochemically induced alkalinization yielded strong, chemically bonded, structurally tailored films with important biological implications and enhanced corrosion resistance.

Micro-arc oxidation (MAO) with bioactive elements incorporated into the electrolyte is a specialized method that can promote the formation of biofunctional ceramic layers containing calcium phosphates and silicate-based phases incorporated into the MgO matrix at the Mg-based alloy surface. This procedure is usually conducted at a high anodic voltage, exceeding MgO’s dielectric breakdown voltage. As a direct consequence, micro-discharges occur at the Mg alloy–electrolyte interface, thereby inducing high-temperature, high-pressure microenvironments. A rapid oxidation is noticed concomitantly with electrolyte solution ions’ (Ca^2+^, PO_4_^3−^, and SiO_4_^4−^) incorporation in MgO under the plasma-assisted reactions, molten-phase solidification, and diffusion phenomenon. The result is a composite ceramic layer containing HAp, amorphous CaP, or magnesium silicates. Lv et al. [70] prepared a ceramic coating on the Mg-Zn-Ca-Ag (ZQ) surface via MAO, containing MgO, Mg_2_SiO_4_, Ca_3_(PO_4_)_2_, CaCO_3_, and Ag_2_O for a concentration of 0.8 wt.% silver (Ag). In addition, the authors varied the Ag content from 0.2 wt.% to 0.8 wt.% in steps of 0.2 wt.% and analyzed the corrosion behavior in the absence of MAO treatment and in vitro antibacterial activity against the *Escherichia coli* (*E. coli*) strain. The potentiodynamic characterization revealed that the much more electronegative value for i_corr_ of 1.87 × 10^−4^ (Acm^−2^) was obtained for 0.8 wt.% Ag in association with an E_corr_ of −1.40 V. Other obtained values for the electrochemical parameters were: i_corr_: 0.2 wt.% Ag—4.70 × 10^−4^ (Acm^−2^); 0.4 wt.% Ag—5.46 × 10^−4^ (Acm^−2^); 0.6 wt.% Ag—5.39 × 10^−4^ (Acm^−2^); E_corr_: 0.2 wt.% Ag—1.42 V; 0.4 wt.% Ag—1.45 V; and 0.6 wt.% Ag—−1.41 V. It can be easily noticed that Ag addition and coating presence significantly reduced the corrosion speed of the Mg-based alloy. Regarding antibacterial activity, the greatest reduction in bacterial colony-forming units (CFUs) was observed with the 0.8 wt.% Ag concentration (99.1%) for the bare Mg-based alloy. However, it is worth noting that the 0.8 wt.% Ag–MAO coated sample also achieved a 97% reduction in CFUs. It was concluded that the best candidate alloy is 0.8 wt.% Ag bare or coated alloy, and that the coating’s complex phase composition was associated with good adhesion and a high-quality, pore-free structure. The main advantage of this method is the ability to obtain an oxide-based matrix enriched with bioactive phases, which provides good protection against corrosive elements and promotes apatite formation, osteointegration, and cell adhesion. However, the MAO procedure, which is a subclass of the PEO method, could inherently induce defect formation due to its high energy requirements. To conclude, this method is suitable for manufacturing complex conversion coatings containing multiple chemical species.

Alkaline treatment followed by conversion mineralization is an innovative surface treatment that is composed of two main parts. Firstly, Mg-based alloys are introduced in an alkaline solution rich in potassium hydroxide (KOH) or sodium hydroxide (NaOH). After that, a porous conversion layer consisting of magnesium hydroxide (Mg(OH)_2_) is formed. Secondly, the alkaline-treated samples are introduced in a physiological fluid that contains Ca^2+^ or PO_4_^3−^ ions. In this way, using a dissolution–reprecipitation route, a CaP-based coating is formed on the Mg(OH)_2_ layer. As a function of process parameters, it could be possible to obtain an amorphous calcium or a crystalline amorphous CaP. You et al. [71] performed a chemical conversion process comprising an alkaline treatment that led to Mg(OH)_2_ formation, followed by a hydrothermal transformation of HAp on the surface of Mg-Nd-Zn-Zr (JDBM). Under the low-temperature effect, the grains of the precursor coating DCPD transformed themselves into spheroidal-like nanocrystals (HA-1h) after 1 h treatment, nanoneedle-like crystals after 6 h (HA-6h), and longer needles after 12 h (HA-12h) of treatment, respectively. A much nobler corrosion character was observed for the HA-12h sample (i_corr_ = 2.06 μA/cm2, E_corr_ = −1.16 V) compared to the uncoated Mg-based alloy (i_corr_ = 4.17 μA/cm2, E_corr_ = −1.16 V). The coating’s biocompatibility was tested in MC3T3-E1 cells using the cell counting kit-8 (CCK-8) assay. It was clear that cells exposed to the HA-1h, HA-6h, and HA-12h extracts showed higher mitochondrial activity and proliferation rates than the control group (uncoated alloy). In addition, the cells exhibited an elongated shape and were attached to the grain edges at HA-1h, and then became polygonal with filopodia and well-formed cell adhesion at HA-12h. The alkaline phosphatase (ALP) activity measurements indicated for HA-12 h a higher value than that of the control group by 29.7%. It was concluded that the developed conversion coating offered an increased corrosion resistance to localized pitting corrosion in conjunction with an improved MC3T3-E1 compatibility and osteoconductivity. The main advantage of this method consists of the improved adhesion, biocompatibility, and protection against corrosive agents offered by the double conversion coating. Unfortunately, challenges such as the instability of the alkaline-derived layer, poor adhesion between layers, and lack of coating uniformity and reproducibility must be addressed. In conclusion, the alkaline treatment followed by conversion mineralization is an efficient and low-cost method to obtain bioactive complex conversion coatings.

The silicate-based conversion coatings (bioglass-type formation) rely on the fact that the bioactive glasses (Bioglass 45S5) could easily form a chemical bond to bone tissue through a hydroxycarbonate apatite (HCA) layer. Usually, this procedure is undergone by Mg alloy immersion in silicate-containing aqueous solution (e.g., sodium silicate (Na_2_SiO_3_) electrolytes supplemented with Ca^2+^ ions). As in the case of other methods, the initial Mg corrosion process leads to Mg^2+^ ion emission and an alkalinization of the environment. Under these conditions, SiO_4_^4−^ undergoes condensation and polymerization. It results in a hydrated silica-rich gel film (Si-OH). As a direct consequence of the Mg alloy dissolution, Mg^2+^ ions are incorporated into the growing layer, leading to the formation of Mg_2_SiO_4_ or amorphous Mg-Si-O compounds. In the presence of Ca^2+^ ions, Ca-Mg silicates are formed, exhibiting a much more complex structure. Li et al. [72] manufactured a coating on the Mg-Al-Zn (AZ31) alloy via a multistage process using phytic acid (PA) conversion, followed by dip-coating and heat treatment. A complex conversion coating consisting of PA/mesoporous 45S5 bioglass (MBG) composite coating was achieved. The authors analyzed the degradation based on immersion tests. The degradation rate for the coated samples was estimated at 0.62 mg per cm^2^ per day on day 16, while the uncoated sample showed a higher value of 2.93 mg per cm^2^ per day. In addition, the coated samples formed an apatite-like structure when exposed to SBF. The bonding strength between the coating and the Mg-based alloy was estimated at 1.52 MPa. It was concluded that the BG-based coating showed a significant increase in corrosion resistance and a strong ability to form apatite, which recommends it as suitable for orthopedic use. The silicate-based coating provides a stable, less soluble coating compared with Mg(OH)_2_, with enhanced biological features due to the controlled release of Si ions that stimulate natural osteogenic activity. On the other hand, careful optimization and control of processing parameters are required to obtain high-quality coatings.

The fluoride–CaP dual conversion coating involves a fluoride treatment followed by CaP deposition. Thus, as a direct consequence, after the initial immersion in HF of the Mg-based alloys, a protective layer of MgF_2_ appears. After that, a mineralization conversion process using CaP-dedicated solutions, saline-buffered solution (SBF), or phosphate-buffered saline (PBS) is performed. On the MgF_2_ layer, a heterogeneous nucleation of CaP based on ion exchange and supersaturation occurs. Su et al. [73] applied a two-step process involving a CaP-deposited coating followed by a fluoride treatment on the Mg-Al-Zn-Mn (AZ60) alloy. The coating composition mainly comprised fluoridated hydroxyapatite (FHA), tricalcium phosphate (TCP), and magnesium fluoride (MgF_2_). The solution pH (10, 11, 12, and 13) and treatment time (0.5, 1, 2, and 6 h) were varied. The electrochemical characterization showed that the highest corrosion resistance was achieved at pH 12 for a treatment time of 2 h (i_corr_ = 0.6 μA/cm^2^), while the lowest corrosion resistance was observed at pH 13 for the same treatment time (i_corr_ = 5.9 μA/cm^2^). Additionally, the much nobler character was observed at pH = 12 and a treatment time of 6 h (i_corr_ = 0.4 μA/cm^2^), while the worst behavior was reported for a treatment time of 0.5 h at a pH = 12 (i_corr_ = 1.6 μA/cm^2^). It can be concluded that processing parameter control is of utmost importance in achieving a high-quality coating. The main advantage of this technique lies in the fact that the MgF_2_ layer acts as a barrier against the action of Cl^−^ ions, while the CaP layer promotes bioactivity, providing a good medium for osteoblast differentiation and proliferation. As stated in ref. [73], the process is influenced by the main working conditions, leading to cracked or brittle CaP films, inhomogeneous coatings, and poor interfacial bonding. To conclude, this complex method is a simple and effective approach that includes MgF_2_ properties and the bioactivity of CaP phases well.

**Figure 4:**
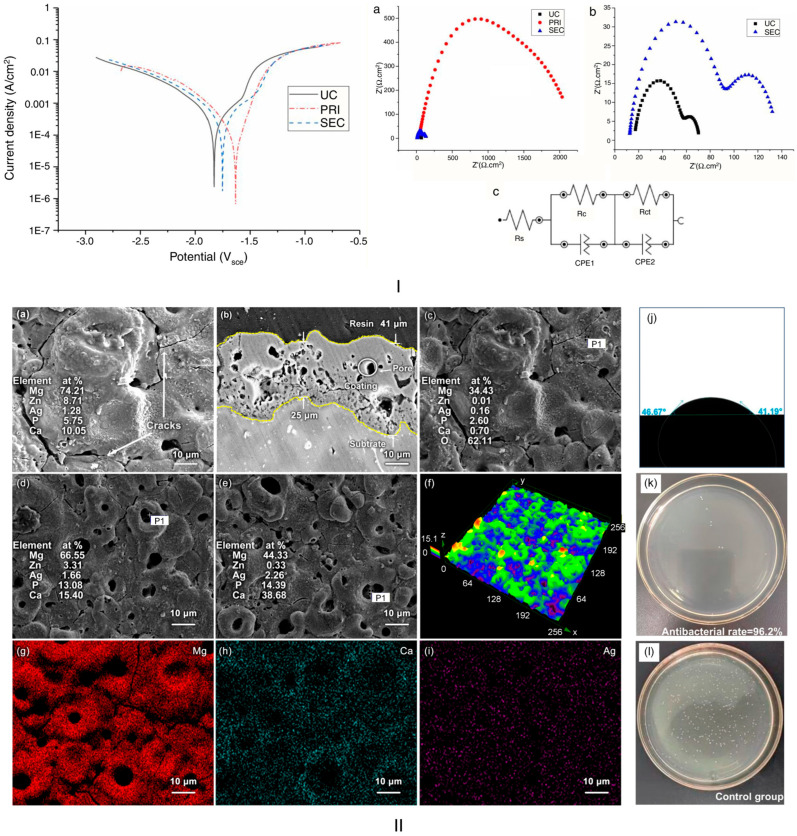
Degradation and biological investigations for bioactive ceramic conversion coatings on Mg-based alloy surface. (**I**) Electrochemical corrosion analysis for pure Mg with CaP conversion coating: Tafel variations; Nyquist graph (**a**) all probes (UC—uncoated, PRI—primary coated, SEC—secondary coating); (**b**) zoom image of UC and SEC samples; (**c**) equivalent electric circuit (Rs—resistance of electrolyte solution, CPE1—constant phase element for high-frequency loop, CPE2—constant phase element for medium-frequency loop, Rct—electron transfer resistance, Rc—resistance of electrodes and contact points) [66]. (**II**) SEM images coupled with EDS analysis in the P1 marked point: (**a**) surface morphology; (**b**) cross-sectional morphology; (**c**) EDS analysis for non-porous MAO coating; (**d**) EDS analysis for MAO coating placed at the micropore edge; (**e**) EDS at the center of micropores; (**f**) 3D view of MAO coating; (**g**) Mg distribution map on the MAO coating; (**h**) Ca distribution map on the MAO coating; (**i**) Ag distribution map on the MAO coating; contact angle and antibacterial activity (*E. coli*) for Mg-Zn-Ca-Ag MAO coated: (**j**) contact angle for MAO coated samples; (**k**) colony plots of antibacterial test on Mg-Zn-Ca-Ag (0.8 wt.% Ag) MAO coated extract; (**l**) control group (negative control). All the images contained in this figure are under CC BY 4.0 or CC BY-NC-ND 4.0 copyright license.

Regarding bioactive ceramic conversion coatings, the field has learned that, in addition to CaP and BG, bioactivity can occur through surface functional groups and the controlled release of ions. These guide protein adsorption, apatite nucleation, and osteoblast differentiation. All ceramic conversion coatings are based on Mg alloy dissolution, which triggers local pH increases, surface supersaturation, and the heterogeneous nucleation of the ceramic phase. This observation leads to the conclusion that, in all cases, coating formation is tied to the Mg degradation process, and achieving perfect control of the process is almost impossible. The highly crystalline coatings are stable, but the amorphous ones are much more bioactive. Regarding the BG-type systems, a second bioactivity route has been introduced based on Si-OH groups and the release of soluble silica. The key challenges are associated with the natural ceramic layers’ brittleness, poor long-term corrosion process, weak adhesion, and difficulty in controlling the phase purity. The most important “take-home” criteria are: it is recommended to use double ceramic systems, to tune phase composition deliberately, to control crystallinity at the nano-scale level, and to incorporate functional ions (F—stability, Si—osteogenesis, and Mg—bioactivity). Figure 4 shows experimental results related to bioactive conversion ceramic coatings for Mg-based alloys.

Table 2 presents a decision summary that complements all the investigated studies and establishes a recommendation for a future biomedical application. To highlight the most important process parameters and establish a systematic integration, the investigations briefly summarized in Decision Table 2 are detailed in Table 3, with particular attention to testing conditions and main study limitations.

### 3.4. Hybrid Conversion Coatings

As noticed in some of the previous studies, hybrid coatings that combine organic and inorganic components are characterized by synergistic interactions at their interfaces. In this zone, the physical and chemical properties of one phase enhance or complement the other material characteristics [74]. Regarding interface bonding mechanisms, it is worth noting that several mechanisms operate at the interface [75]. The first one is chemical bonding, which is provided by different functional groups of the polymers (carboxyl, hydroxyl, and amine) that can react with phosphate or hydroxide groups on the ceramic layer surface. In this way, covalent or coordinate bonds are formed. Another mechanism is the physical interlocking due to porosity or surface roughness, which is present in the ceramic layer and enables mechanical interlocking with the polymer chains. A polymer infiltration effect in the micro- and nano-pores occurs, and improved mechanical properties and resistance to cracking are expected. Last but not least, some hybrid coatings are characterized by electrostatic interactions between the ceramic and polymeric components, a uniform coverage, and a low amount of micro-defects, which act as trigger points for corrosion initiation [76].

One of the most important synergistic effects is barrier enhancement. The inorganic phase forms a physical barrier to ion penetration, while the polymeric phase seals surface defects, thereby reducing corrosion rates in Mg-based alloys. Another aspect is the controlled degradation and ion release resulting from the incorporation of bioactive ceramic components, which can enhance osteointegration. By adding a polymeric phase, ion flux can be modulated, preventing toxic ion accumulation. In some cases, it is possible to incorporate functional molecules, such as growth factors or drugs, into the polymeric component, while the inorganic phase provides mechanical support and delivers bioactive ions [77].

Analyzing and clearly understanding the interface bonding and synergistic mechanisms are important when multifunctional conversion coatings are developed. By controlling and optimizing the chemical functionality, ceramic porosity, and polymer molecular weight, a precise control over mechanical properties, bioactivity, and corrosion rate is facilitated. In this way, the gap between laboratory analyses and the clinical performance of Mg-based alloys could be bridged.

### 3.5. Industrial Preparation and Clinical Translation of Conversion Coatings

As shown in the previous sections, the conversion coatings on the Mg-based alloy surface proved to be highly efficient at the laboratory scale. In addition, their industrial translation is limited not only by scale-up challenges but also by process complexity and economic feasibility.

One of the most important challenges is cost-effectiveness across all manufacturing steps (e.g., raw materials, processing parameters, and post-treatment requirements). Usually, high-performance conversion coatings include expensive elements, such as rare earth metals, which increase the final cost of the Mg-based alloy [35]. Coating techniques such as MAO/PEO or hydrothermal treatment are characterized by applying special conditions, such as high working temperature, pressure, and electric voltage, which result in a high energy input per unit area, making these methods hardly applicable at an industrial level. Another challenge foreseen is that laboratory-performed immersion or deposition processes require long reaction times, which limit their large-scale application. Additionally, conversion coatings are followed by waste compounds that require treatment to be in line with the new environmental regulations [78,79]. One can immediately notice that low-cost chemistry, in conjunction with process simplification, should be accomplished.

Important problems can occur when scaling from a laboratory to an industrial level is needed (process amplification and reproducibility). Coating film uniformity is difficult to achieve on large components due to local chemical variations, temperature gradients, and fluid dynamics. During the chemical conversion processes, the bath composition is highly influenced by ion depletion and byproduct accumulation, which affect coating quality and require continuous monitoring and adjustment [80]. Last but not least, small fluctuations in ion concentration, pH, and temperature are usually linked to coating structure quality, by changing the local crystallinity or inducing unwanted porosity, making the industrial process less robust. Thus, as a direct consequence, the process standardization, closed-loop control system, and real-time monitoring are of utmost importance.

A major challenge in large-scale production is achieving a uniform coating on implants with complex geometries. Firstly, conversion coatings obtained after the immersion procedure exhibit limited accessibility and often result in non-uniform growth, which can initiate pitting corrosion. Then, H_2_ gas evolution from Mg-based alloy corrosion leads to a microenvironmental shift, with local disruption of the coating formation. Regarding additive manufacturing technologies, the Mg implants produced using them are porous or contain channels, and the penetration and adhesion of coatings are very difficult to control. Advanced methods such as electrophoretic deposition-assisted conversion or hybrid techniques could be applied, but validation for industrial-scale application remains limited [81].

Regarding clinical translation, it should be noted that conversion coatings must be compatible with existing medical manufacturing systems [13,82]. Surface preparation steps should be scalable and standardized because they significantly contribute to coating adhesion and reproducibility. Additionally, conversion coatings must be compatible with the sterilization methods used in medicine without degrading the implant. The lack of good manufacturing practices and medical regulations restricts the large-scale application of conversion coatings.

Some strategic directions for a successful industrial translation are put forward, including the application of low-cost, environmentally friendly chemistry; high-throughput and energy-efficient techniques; and advanced process control. In addition, hybrid and multilayer strategies and the standardization of coating protocols are worth mentioning as necessities for a large-scale application of the conversion coating.

### 3.6. Coupling Relationship Between the Mechanical and Corrosion Properties of the Conversion Coatings

It is of utmost importance to establish a link between Mg-based alloy corrosion resistance and mechanical performance because, usually, in the case of complex physiological environments, such as load-bearing conditions, these features are not independent. Based on the coupling phenomena, it could be established if the coated Mg-based alloy behaves as expected [83,84].

First of all, under in vivo conditions, Mg-based implants must overcome cyclic mechanical loading, which generates localized stress concentration in the conversion coating layer. In the case of brittle ceramic layers, such as MAO-prepared or phosphate-based coatings, microcracks could occur [85,86]. A direct consequence is the accelerated crack propagation if the coating contains interfacial regions or defects. Another aspect that is worth mentioning is the possibility of biological fluids rich in Cl^−^ ions to penetrate and to trigger the localized corrosion process. Analyses of PEO/MAO coatings revealed microstructural heterogeneity and porosity that act as stress concentrators, making the coating prone to cracking under bending or tensile loads [87,88,89].

Microcracks result from residual stresses that occur during conversion coating formation, from elastic mismatch between the Mg alloy and the protective layer, or from external mechanical loading. An important consequence of microcracks consists of barrier integrity loss, the appearance of localized galvanic cells between the coated regions of the surface, newly exposed Mg substrate, and last but not least, an increased gas release and pitting corrosion initiation [17,84].

It is well known that the low elastic modulus of polymeric-based conversion coatings offer better flexibility and increased crack resistance, but, concomitantly, they exhibit a reduced corrosion barrier protection. On the other hand, ceramic coatings have better corrosion barrier efficiency but are brittle in nature. Thus, an optimal conversion coating must establish an equilibrium between structural integrity and mechanical compliance to maintain an increased corrosion resistance under natural complex physiological loads.

Some research studies [48,57] previously presented described the application of self-healing and adaptive coating systems. They consist of incorporating corrosion inhibitors into coatings or microcapsules, which allows controlled release upon crack formation (inhibitor-based self-healing) [90,91]. A new trend is foreseen: smart polymers that can seal microcracks or swell upon fluid ingress [92,93]. Last but not least, sol–gel and hybrid systems that could enable dynamic re-bonding or re-condensation reactions are also important, but this research field is still in its early stages [94,95]. Unfortunately, these interesting approaches are limited to the proof-of-concept stage, with limited validation in real in vivo conditions [86,96,97].

In the medical field, for the Mg-based implant domain, coatings must resist cyclic loading without initiating cracking; if damaged, they should exhibit self-healing properties, and the degradation process must be controlled and uniform, even if the implant fails mechanically. To investigate these types of phenomena, corrosion-fatigue tests, in situ mechanical loading in physiological fluids, and fracture-corrosion interactions approaches must be followed.

### 3.7. Quantitative Performance Evaluation and Selection Criteria for Conversion Coatings

By analyzing some of the literature studies presented in the review paper, an important limitation was identified. A standardized quantitative evaluation framework must be proposed, as many investigations rely on qualitative or isolated performance indicators. It was shown that conversion coating behavior is multi-parametric, encompassing simultaneous effects on corrosion resistance, biological response, mechanical integrity, ion release, and degradation kinetics.

To solve this gap, the conversion coating performance should be evaluated based on a measurable indicator list. Firstly, corrosion behavior could be quantified by the corrosion current density, degradation rate, and hydrogen evolution rate. Then, mechanical performance could be assessed via elastic modulus, adhesion strength, hardness, and resistance to crack initiation. Regarding the biological response, quantitative parameters such as cell viability, in vivo bone-implant boundary analysis, and osteogenic marker expression should be adopted. In addition, functional properties, such as ion release kinetics and coating integrity, will be critical for long-term analysis in the development of a bone implant.

A multi-criteria performance index (PI) could be proposed as: PI = w_1_A + w_2_B + w_3_C + w_4_D, where A, B, C, D are normalized values for corrosion phenomenon, mechanical properties, biological performance, and functional attributes, and w_i_ (i = 1–4) are weighting factors generated by different approaches. To conduct a comparison analysis, all quantities should be normalized to a dimensionless scale based on clinical thresholds and literature benchmarks [98,99,100]. Additionally, the relative weighting of the above-mentioned criteria has to be application dependent. For example, in the case of orthopedic implants, biocompatibility and controlled degradation are of utmost importance, while for cardiovascular stents, a strict analysis of corrosion rate and mechanical stability must be completed.

Unfortunately, despite the high potential of the implementation of such a promising quantitative system, limitations are seen due to the lack of standardized testing protocols unanimously accepted by all scholars, reproducible data sets between the literature studies, and long-term in vivo quantitative data, as will be underlined in the next section of the review paper.

It is generally accepted that future efforts must be channeled towards a harmonized evaluation methodology and the integration of multi-scale experimental data into robust decision-making models, such as Digital Twins.

## 4. Biological Aspects Related to Conversion Coatings Used to Modify the Surface of Mg-Based Alloys

It is widely accepted that Mg alloy biocompatibility must be discussed in correlation with degradation process kinetics. As emphasized by Hornberger et al. [14] and Kumar et al. [101], and as presented in Section 3 of this review paper, during the Mg-based alloy corrosion process, increased alkalinization of the medium, hydrogen gas evolution, and osmotic stresses are induced. All of these factors have important implications for cell viability and tissue regeneration [102,103]. Adequate conversion coatings serve as a biointerface zone, acting as a corrosion barrier [104,105,106].

Ceramic conversion coatings based on metals have a beneficial effect on degradation stabilization, by reducing the corrosion current density values and hydrogen evolution. Some studies correlated these findings with improved cytocompatibility by generating a physiological pH value [36,38,52]. In refs. [1,107,108,109], it is stated that to improve the biological performance of an inert coating, bioactive elements should be added or the coating should be functionalized with different chemical groups. Various studies [25,26,43] showed that PEO coatings can support apatite nucleation, but when a CaP-rich solution is not used, the coating shows limited bioactivity.

Polymeric conversion coatings are much more suitable for the biological integration of an implant because, in some cases, such as PDA or gelatin–chitosan [55,56,57], they exhibit biofunctional surfaces with increased protein adsorption, cell adhesion, and proliferation, as well as important antibacterial or bioactive properties, when Zn, inhibitors, or growth factors are included. Refs. [110,111,112,113] proved that polymeric layers could control the local alkalinization profile and decrease the cytotoxicity associated with Mg-based dissolution during the early stages of the process. To increase the life span of the polymeric coatings, a hybridization with ceramic layers is recommended [114].

Bioactive ceramic coatings based on CaP compounds or bioglasses are some of the best candidates when bioactivity is the most important feature [63,115,116,117]. As indicated in refs. [30,65,66,68], increased osteoconduction combined with apatite formation and good cell differentiation, proliferation, and adhesion was evident. Hydrothermal HAp coating improved a high amount of cell viability concomitantly with a significant amount of H_2_ gas emission [32], while Sr-doped HAp activates important regenerative signaling pathways [118]. As stated before, attention should be devoted to a possible delamination process and the brittle character of the ceramic.

Table 4 presents the main biological pathways activated by different conversion coatings on Mg-based alloys. The osteogenic signaling pathways are the BMP/Smad pathway, which promotes osteoblast differentiation and bone matrix deposition; the Wnt/β-catenin pathway that is critical for osteoblast maturation and proliferation; and the MAPK/ERK pathway dedicated to cell proliferation and differentiation in relation to the surface chemistry and coating topography. For the angiogenic signaling pathways, one should consider the VEGF/VEGFR pathway, which promotes endothelial cell migration and new blood vessel formation, and the PI3K/Akt pathway that enhances endothelial cell survival, tube formation, and migration. As an overall observation, the conversion coatings generate a surface medium that is topographically, chemically, and biologically conducive to cell proliferation and attachment, while simultaneously mitigating the adverse biological response to uncoated Mg-based alloys.

**Figure 5:**
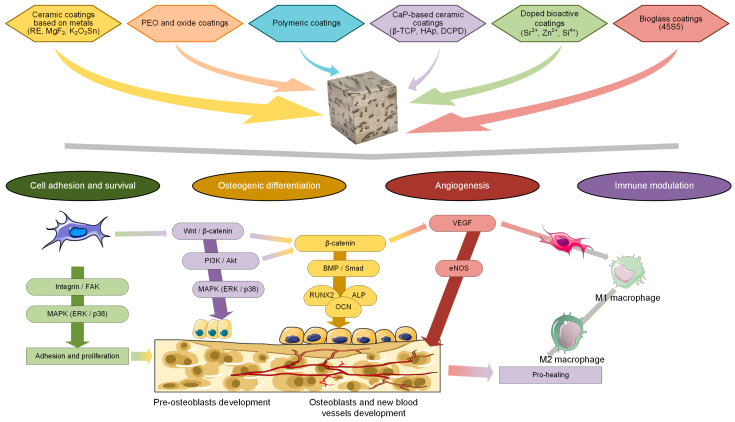
Schematic representation of the biological signaling pathways involved in osteogenesis, angiogenesis, and immune host response modulation in relation with the conversion coatings for Mg-based alloys. This figure was generated using images adapted from Servier Medical Art (https://smart.servier.com, accessed on 21 March 2026), licensed under CC BY 4.0.

Information is provided on the relationships between coating particularities and biological pathways linked to osteogenesis, angiogenesis, and the immune host response, based on the case of Mg alloys (Figure 5).

A detailed analysis of cellular signaling pathways must be conducted considering the relationships among specific ionic species, dose-dependent cellular responses to the main molecular mechanisms, and ion release kinetics.

Strontium-containing conversion coatings (Sr-doped Ca-P and MAO films) can modulate bone remodeling through coupled osteoblast–osteoclast regulation. It is well known that Sr^2+^ ions activate the CaSR, triggering downregulation of the MAPK/ERK and Wnt/β-catenin signaling pathways [119]. As a direct consequence, osteogenic markers such as ALP, RUNX2, and OCN are upregulated, stimulating osteogenesis. Simultaneously, Sr^2+^ ions downregulate the RANKL/OPG ratio by inhibiting NF-kB-mediated osteoclast differentiation [120]. At low to moderate Sr^2+^ concentrations (0.1–1 mM), osteoblast proliferation and differentiation are sustained, whereas at high concentrations (>2–3 mM) cytotoxic effects may occur, leading to perturbed cellular homeostasis [121].

Another important aspect worth mentioning is related to silicon-based conversion coatings (bioactive glass-derived films), which can release soluble silicate species that play an important role in osteogenesis and angiogenesis. Si^4+^ ions stimulate Wnt/β-catenin and BMP-2/Smad signaling by enhancing the expression of ALP, RUNX2, and COL1A1. In addition, Si^4+^ ions promote endothelial cell activity via VEGF signaling and sustain proper angiogenesis [122,123]. At a low concentration of Si^4+^ ions (10–50 μM), osteoblast and endothelial cell proliferation and differentiation are sustained. At higher ion concentrations (>100 μM), increased oxidative stress may occur, with a direct effect on cell viability [124].

Mg^2+^ ions are regulators of osteogenic differentiation and early-stage cell adhesion. They activate integrin-mediated signaling and downstream FAK/PI3K/Akt pathways, improving the osteoblast differentiation [125]. In addition, Mg^2+^ modulates Wnt/β-catenin signaling, promoting an increased expression of ALP and RUNX2 osteogenic markers. Also, magnesium ions polarize macrophages toward an M2 (anti-inflammatory) state, supporting tissue regeneration [126]. At moderate concentration (2–10 mM), locally applied, the proliferation and differentiation of osteoblasts are enhanced, whereas at higher concentrations, alkalinization, osmotic stress, and cytotoxicity are observed [127].

Ca^2+^ ions play an important role in bone formation by activating the CaSR and triggering MAPK/ERK and PKC pathways, which sustain osteogenesis. Additionally, calcium contributes to an equilibrium between osteoblasts and osteoclasts via RANKL/OPG signaling. At moderate levels, it enhances matrix mineralization and osteogenesis, whereas at high concentrations, it may be linked to apoptosis and cell stress [128].

Usually, in a conversion coating, multiple ions, such as Mg^2+^, Ca^2+^, Sr^2+^, and Si^4+^, are released simultaneously, and synergistic or antagonistic interactions can form. It is well known that Mg^2+^ ions enhance cell adhesion and promote early osteogenesis, but they also lead to environmental alkalinization [21,129]. Mg^2+^ and Ca^2+^ ions have complementary and synergistic effects. Mg^2+^ ions sustain cell adhesion and proliferation, while Ca^2+^ ions enhance mineralization and matrix maturation. Both types of ions support bone remodeling, but excessive co-release amplifies local pH shifts and ionic imbalances, with a visible effect on cell viability. Ca^2+^ and Sr^2+^ ions synergistically regulate bone remodeling via CaSR activation, while Si^4+^ and Sr^2+^ improve overall tissue regeneration by coupling the osteogenesis (Sr) and angiogenesis (Si). Excessive cumulative ion concentrations can have negative effects, including altering local pH, disrupting ionic homeostasis, and triggering cytotoxic or inflammatory responses [130,131].

The temporal evolution of ion release is characterized by an initial burst release with impact on early inflammation or stress responses, followed by a sustained release phase, which supports the long-term osteogenic processes, and a late-stage degradation phase that is directly linked to remodeling and integration. Although these promising effects are well known, there are still limitations regarding a quantitative correlation between ion concentration and specific signaling pathway activation, insufficient analysis of crosstalk between different signaling pathways under multiple ion action, and limited studies with dose–response variations with protein/gene expression.

The field has learned that ceramic conversion coatings enhance cell survival through PI3K/Akt and MAPK pathways, concomitantly with a stabilization of the corrosion rate [13]. Also, the PEO coatings activate the integrin-FAK signaling pathway and increase cell adhesion [132]. Polymeric conversion coatings have a beneficial effect on early-stage cell responses, as evidenced by FAK and PI3K/Akt activation [133,134]. Bioceramic coatings trigger osteogenesis based on Wnt/β-catenin, BMP/Smad, and CaSR pathways [135,136,137,138]. In the case of certain dopants, angiogenesis could be stimulated via VEGF signaling [130], [139]. Hybrid coatings are characterized by a cumulative effect consisting of sequential activation of cell survival, differentiation, and proliferation [74]. Last but not least, Mg^2+^ induces a local host immunomodulatory response [140].

Even though many of the studies presented in this review focus on in vitro corrosion behavior and cytocompatibility, it is important to note that in vivo animal studies show that the biological performance of Mg-based alloys with a conversion coating is characterized by time-dependent interactions among tissue healing, host immune response, and degradation kinetics.

Regarding the long-term degradation behavior, it is well known that in vivo degradation is a dynamic process highly influenced by the physiological medium. The animal studies showed that degradation kinetics are strongly dependent on blood flow and tissue type, with accelerated Mg-based alloy degradation observed in highly vascularized zones [2], whereas a much weaker effect was observed in the cortical bone region [141].

Some long-term investigations [142,143] performed on murine animals or rabbits proved that a controlled degradation is essential in maintaining mechanical integrity during the bone healing process, favoring the gradual replacement of the coated Mg-based alloy with new bone. However, the main reported side effects were due to excessive hydrogen gas accumulation and cavity apparition, and a local alkalinization that can generate cell death. As a direct consequence, the conversion coating should favor temporal synchronization with the natural tissue-healing rate and increase corrosion resistance.

As stated before, the release of Mg^2+^ ions is associated with increased bone formation and an enhanced mineralization process [144]. In ref. [142], it was shown that coated Mg-based implants supported early-stage cell adhesion concomitantly with the appearance of fibrous tissue, thereby providing initial stabilization of the implant. Another study [143] demonstrated that rapid, increased degradation of the implant led to reduced bone-to-implant contact, affecting long-term fixation. An important conclusion is that structural stability should always be correlated with osteointegration.

Regarding the host immune response, Mg-based alloys can elicit localized inflammatory responses, characterized by macrophage infiltration and transient inflammation [145]. Also, the intensity of the immune response is directly linked to the rapid or slow degradation profile. A recent in vivo study [146] showed that properly engineered conversion coatings can reduce toxicity and immunogenicity and control the amount of Mg^2+^ ions released.

Some current limitations must be underlined, such as the insufficiency of long-term in vivo studies for coated Mg-based alloys, the scarcity of correlations between in vitro degradation models and in vivo expected outcomes, limited information regarding the chronic immune response and foreign body reactions, as well as the lack of standardized animal models and evaluation protocols for performing literature comparisons.

To summarize, the main challenges associated with the biological performance of conversion coatings are: uncontrolled degradation–biological mismatch, difficult control of biological signaling, limited long-term stability of conversion coating, equilibrium between bioactive character and corrosion resistance of the conversion coating, insufficient studies on immunomodulatory and angiogenic evaluation, and the lack of standardized measurements. The main “take-home” criterion is that a perfect conversion coating has to degrade at a dissolution rate matched to the healing process, to activate the correct biological pathways to trigger the regeneration of the bone or vascular defect, and to maintain mechanical and interfacial properties.

**Table 4:** Biological pathways activated by some of the most commonly used conversion coatings on Mg-based alloys.

Coating	Key Biological Triggers	Signaling Pathway	Molecular Markers	Cellular Response	Contribution to Bone Formation	Ref.
Ceramic conversion coating based on metals	Mg^2+^ ions ‘release, reduced alkalinization	PI3K/Akt; MAPK	Akt, ERK1/2, p38	↑Survival ↓Apoptosis	Indirect osteogenesis	Singh et al. [13]
PEO/oxide coatings	Surface roughness, ion exchange	MAPK, Integrin-FAK	FAK, ERK	↑Adhesion and proliferation	Early osteointegration	Fattah-alhosseini et al. [132]
Polymeric coatings (PDA, PPy)	Functional groups, protein adsorption	PI3K/Akt, FAK	Akt, vinculin	↑Adhesion and proliferation	Early cell anchorage	Harati et al. [133], Li et al. [134]
LbL coatings	ECM-like chemistry	Integrin-FAK, PI3K/Akt	FAK, paxillin	↑Spreading ↓Inflammation	Early tissue formation	Kunjukunju et al. [62]
Bioactive ceramic coatings (CaP, HAp, DCPD)	Ca^2+^/PO_4_^3-^	Wnt/β-catenin, BMP/Smad/CaSR	RUNX2, VEGF	↑Differentiation mineralization	Direct osteoinduction	Li et al. [135], Tang et al. [136]
Doped bioactive coatings (Sr^2+^, Zn^2+^, Si^4+^ ions)	Sr^2+^, Zn^2+^, Si^4+^ release	Wnt/β-catenin, BMP, VEGF	RUNX2, VEGF	↑Osteogenesis, angiogenesis	Accelerated bone formation	Mao et al. [130], Amaravathy and Kumar [139]
Bioglass coatings	Si^4+^, Ca^2+^ release	VEGF, Wnt/β-catenin	VEGF, eNOS	↑Angiogenesis	Vascularized bone formation	Crush et al. [137], Saffarian Tousi et al. [138]
Hybrid multilayer coatings	Sequential ion release	PI3k/Akt, MAPK, Wnt, BMP, VEGF	Akt, RUNX2, VEGF	Balanced proliferation and differentiation	Optimal regeneration	Khatun et al. [74]
System-level (immune response)	Mg^2+^ release	M1→M2 polarization	IL-10, TGF-β, CD206	↓Inflammation	Regenerative environment	Bessa-Goncalves et al. [140]

↑—increase; ↓—decrease; PI3K/Akt—Phosphoinositide 3-kinase/Protein Kinase B; MAPK—Mitogen-Activated Protein Kinase; ERK1/2—Extracellular Signal-Regulated Kinase; p38—38 kilodaltons; FAK—Focal Adhesion Kinase; ECM—extracellular matrix; BMP—bone morphogenetic protein; Smad—direct translator BMP; RUNX2—Runt-related transcription factor 2; VEGF—Vascular Endothelial Growth Factor; eNOS—endothelial Nitric Oxide Synthase; M1—pro-inflammatory macrophage polarization; M2—anti-inflammatory macrophage polarization; IL–10—Interleukin-10; TGF-β—Transforming Growth Factor-beta; CD206—Cluster of Differentiation 206; CaSR—Calcium-Sensing Receptor.

## 5. Conclusions, Future Trends, and Key Outstanding Questions for the Conversion Coatings on Mg-Based Alloys Domain

From the previous review paper sections, the main conclusion is that conversion coatings enhance the corrosion resistance of Mg-based alloys, thereby addressing an important limitation of magnesium behavior in physiological media. Graphical variations and numerical values of the corrosion current density and corrosion potential show that the coated samples exhibit much more electronegative and electropositive values, respectively, across all the investigated literature studies. From this observation, it is evident that conversion coatings act as barriers that reduce the corrosion rate of Mg-based alloys. In addition, for the bioactive conversion layers, improvements in biocompatibility are observed. These types of conversion coatings are suitable for orthopedic use since they promote cellular-specific pathways dedicated to osteogenesis and angiogenesis. Additionally, it can be observed that solvothermal and hydrothermal strategies yield crystalline, dense coatings, whereas electrochemical methods enable precise control over porosity, thickness, and the incorporation of bioactive ions. The combination of conversion coating methods into so-called “hybrid methods” leads to a significant reduction in corrosion rate and an increased biological response.

From Table 3, it can be seen that for rare earth and stannate-based conversion coatings, the corrosion analysis was conducted under non-physiological conditions, using NaCl-based electrolytes at room temperature, which could limit their application in the medical field. In the modern approach, cases such as Ca-P, MgF_2_, MAO/PEO, and polymer-assisted conversion coatings are expected to provide enhanced control over coating composition, functionality, and microstructure. Also, Ca-P and biomimetic conversion coatings deposited at 37 °C in SBF demonstrated enhanced osteogenic potential and corrosion control, while hybrid systems generated synergistic effects, providing both barrier protection and biofunctional interfaces.

An important limitation, namely the lack of a systematic integration of process parameters, was noted, as coating manufacturing is sensitive to solution chemical formula, pH, and treatment duration. However, different approaches were presented (Table 2 and Table 3). It was observed that engineering-oriented testing was performed in a NaCl solution at room temperature, while biomedical testing was conducted in SBF at 37 °C. In addition, the literature uses different evaluation methods, and drawing a clear conclusion about their advantages and disadvantages is difficult. Also, a quantitative correlation between process parameters, coating structure, and long-term performance should be addressed by considering the relation between the corrosion process and the material’s biological response. The standardization of analysis methods should include standardized coating procedure parameters (solution chemical composition, pH, temperature, and treatment time), the clear classification and use of electrolytes, the normalization of corrosion parameters to permit comparison across studies, and a hierarchical evaluation protocol for hybrid coatings.

Future trends should address the possibility of increasing the conversion coating thickness, but an equilibrium should be established in relation to the decreased adhesion and brittle character of the coatings. Porous coatings are well known to favorize bone in- and on-growth, while micro- and nano-scale surface topographies enhance the cellular response. Supplementary doping with ions such as Ca^2+^, Zn^2+^, Si^4+^, or even Mg^2+^ should be further investigated, as they promote osteogenesis, but the exact cellular pathways are not yet entirely established for each ion case. Hybrid techniques, such as functionalization methods or the addition of bioactive molecules, should be further analyzed, given their high potential for generating bioactive coatings. An important future direction is the development of stimuli-responsive coating systems that become adaptable and exhibit on-demand functionality [147]. These special coatings must respond dynamically to local changes in the medium, such as pH variation, ion concentration, corrosion initiation, or mechanical damage. For example, in a pH-responsive system, the release of bioactive ions or corrosion inhibitors in acidic zones associated with localized degradation processes could decrease corrosion rates and enhance tissue repair. As previously described, self-healing coatings with micro/nanocontainers could restore microcrack formation and, in a facile way, interrupt the mechanochemical degradation cycle [148]. Also, smart hybrid coatings containing sol–gel matrices, polymers, or bioactive ceramics proved to modulate the ion release kinetics in accordance with the different healing stages of the natural tissue. Despite these interesting developments, the field of intelligent coatings is still at the proof-of-concept stage, and a lack of long-term in vivo investigations and mechanical load analyses is noted [149].

As previously noted, there are important challenges that must be addressed before a possible clinical translation of Mg-based implants with biocompatible conversion coatings. These aspects include: a rapid and non-uniform corrosion process linked to a premature loss of mechanical support; a controlled ion release to avoid inflammatory and cytotoxic reactions; coating stability and adhesion to prohibit film delamination or crack initiation; patient-specific variability, which should include age, metabolic activity, and overall health conditions; and last but not least the integration of the multifunctionality concept by establishing a stable energy state between mechanical integrity, corrosion control, and bioactivity, to ensure an ideal synchronization between Mg alloy degradation and new bone formation.

**Figure 6:**
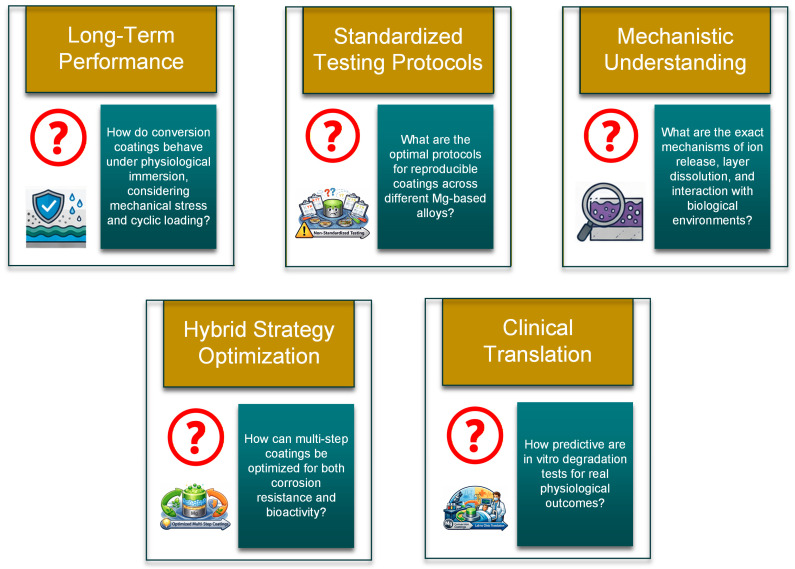
Key outstanding questions box.

Considering the above-mentioned observations, there are still important key outstanding questions (Figure 6). Today, there are just a few studies that have investigated the long-term performance of conversion-coated Mg alloys under mechanical stress and cyclic loading coupled with immersion tests, so this direction must be further explored. The lack of standardized testing protocols across Mg alloys leads to the questionable reproducibility of the measurements. The physical, chemical, and biological mechanisms should be well understood to perform a connected analysis of phenomena such as coating dissolution, ion release, and biological response, as they may occur simultaneously, and a mathematical model must be developed to explain and understand these complex behaviors fully. Optimizing hybrid methods to achieve effective corrosion protection while enhancing bioactive action remains an important challenge for the future. Last but not least, a translation route from laboratory analyses to industrial scale remains a pressing concern. Finding answers to these questions will ensure the clinical translation of conversion coating methods from the laboratory bench to clinical beds.

## Figures and Tables

**Table 1:** Effect of conversion coating on the degradation process and biocompatibility/bioactivity in Mg-based alloys.

Conversion Coating Method	Mg Alloy	Coating	Degradation Analysis Results	Biocompatibility/Bioactivity	Ref.
Sol–gel	Mg-Zn-RE-Zr (ZE41)	Silica (SiO_2_) based sol–gel matrix	Electrochemical analysis $\mathrm{Barrier}\;\mathrm{resistance}:\;\mathrm{The}\;\mathrm{total}\;\mathrm{resistance}\;(R_{p}$) of the coated alloy increased by approximately three orders of magnitude compared to the bare substrate after 24 h of immersion in 3.5% NaCl solution Corrosion parameters: i_corr_/accumulated CR = 26.08 μA/cm^2^/812.5 μg/cm^2^ (coated samples) compared to 84.41 μA/cm^2^/285.4 μg/cm^2^ (bare substrate) Sintering effect: Higher impedance values were obtained for coatings sintered at 200 °C compared to 150 °C	No in vitro or in vivo studies were reported	Lopez et al. [18]
Sol–gel combined with dip-coating	Mg-Al-Zn (AZ31B) and Mg-Al-Zn-Mn (AZ91D)	Two types of silica sols were prepared: one without colloidal silica particles (TG sol) and one containing colloidal silica particles (MTL sol)	In vitro tests in SBF (hydrogen evolution, pH variation, and potentiodynamic polarization curves) Performance Variation: The results showed that performance depended heavily on the alloy and coating composition Blocking Capability: Coatings prepared with the MTL sol on AZ91D alloy were able to effectively block the degradation of the substrate for 8 days of immersion in SBF (uncoated 1.63 mL/cm^2^/day; coated 0.019/1.31 mL/cm^2^/day for MTL and 0.177/1.27 mL/cm^2^/day for TG), while the AZ31B-TG coatings maintained stability for only 3 days (uncoated 1.37 mL/cm^2^/day; coated 0.153/1.34 mL/cm^2^/day for MTL and 0.163/1.57 mL/cm^2^/day for TG)	Mg(OH)_2_ and HAp were identified as corrosion products precipitating on the AZ31B samples, which were not observed on the AZ91D samples	Castro and Duran [19]
Hybrid organic–inorganic sol–gel	Mg-Al-Zn (AZ31)	A hybrid silica-zirconia-based sol–gel coating (modified with organo-functional groups to increase flexibility and reduce cracking) was doped with 8-HQ	Electrochemical Impedance Spectroscopy (EIS) EIS showed that the inhibitor-doped system effectively maintained a high barrier resistance over longer immersion periods compared to the reference (undoped) sol–gel, which showed a rapid decline in protection as the electrolyte penetrated the coating (resistance of the sol–gel film R_SG_: 7.231 × 10^3^ Ωcm^2^ (before hydrolysis); 20.13 × 10^3^ Ωcm^2^ (after hydrolysis); 4.701 × 10^3^ Ωcm^2^ (undoped)) Mechanism**:** The 8-HQ acts by forming complexes at the sites where corrosion initiates, effectively “blocking” the micropores and micro-defects in the sol–gel film	No in vitro or in vivo studies were reported	Galio et al. [20]
PEO	Mg-Al-Zn (AZ31)	The PEO coating was doped with TCP particles. A porous, ceramic-like layer that serves for the nucleation and growth of apatite compounds upon immersion in biological fluids was achieved	EIS and potentiodynamic polarization analysis Polarization resistance (R_p_): The addition of TCP particles to the PEO electrolyte significantly improved the barrier properties. The R_p_ values increased from 18.66 kΩcm^2^ for the PEO-coated samples to about 43.20 kΩcm^2^ for the TCP-modified PEO-coated samples Corrosion current density: The TCP-modified coating exhibited a reduction from 0.155 μA/cm^2^ to 0.0638 μA/cm^2^, showing a significant improvement in the corrosion inhibition of the AZ31 substrate in the SBF environment	The TCP-modified PEO coating facilitates the formation of a HAp layer on the surface during immersion in SBF	Attarzadeh et al. [22]
Anodizing method	Mg-Al-Zn (AZ31)	Anodic oxide coatings. The influence of different concentrations of sodium silicate, sodium hydroxide, and sodium fluoride in the electrolyte solution to optimize the coating thickness and barrier integrity was investigated	Potentiostatic polarization and EIS $\mathrm{Corrosion}\;\mathrm{Current}\;\mathrm{Density}:\;\mathrm{The}\;\mathrm{results}\;\mathrm{demonstrated}\;\mathrm{that}\;\mathrm{an}\;\mathrm{optimized}\;\mathrm{electrolyte}\;\mathrm{composition}\;\mathrm{significantly}\;\mathrm{reduced}\;\mathrm{the}\;\mathrm{corrosion}\;\mathrm{current}\;\mathrm{density}.\;i_{\mathrm{corr}}\;\mathrm{for}\;\mathrm{the}\;\mathrm{anodized}\;\mathrm{sample}\;\mathrm{was}\;\mathrm{about}\;1.6\times 10^{-6}\;{A/\mathrm{cm}}^{2}$$,\;\mathrm{compared}\;\mathrm{to}\;5.0\times 10^{-5}\;{A/\mathrm{cm}}^{2}$ for the bare AZ31 alloy Impedance Performance: The total impedance at low frequencies for the coated samples showed an improvement of nearly two orders of magnitude compared to the uncoated samples, confirming that the electrolyte chemistry had an important influence on the porosity and quality of the anodic layer	No in vitro or in vivo studies were reported	Hassein Mousavian and Tabaian [25]
Chemical conversion based on immersion in a solution that facilitates the spontaneous precipitation of CaP minerals on the alloy surface	Mg-Al-Zn (AZ31)	CaP chemical conversion coating	Immersion tests Hydrogen Evolution**:** The CaP-coated Mg alloy samples showed a significant reduction in H_2_ gas evolution. While bare AZ31 exhibited a rapid initial rate of H_2_ evolution, the coated samples effectively delayed the onset of rapid corrosion pH Stability**:** The coating provided better control over the local pH increase; the bare alloy caused the surrounding fluid pH to rise quickly due to Mg(OH)_2_ formation, whereas the CaP coating acted as a kinetic barrier	MG-63 (human osteosarcoma cell line) It was found that the micromorphology of the coating is an important factor that influences its biocompatibility**.** Cells exhibited better adhesion and differentiation on specific CaP morphologies that provided a larger surface area and better anchoring sites for cells	Hiromoto and Yamazaki [30]
Chemical conversion based on immersion in HF	Mg-Nd, Mg-Zn	MgF_2_ coating. The coating is formed as a secondary result of the innovative manufacturing route (hybrid weaving/casting-infiltration), resulting in a conformal layer that follows the complex geometry of the porous implant (implants: Mg-Nd_I, Mg-Zn_I; reference: Mg-Nd_R, Mg-Zn_R)	Immersion test Mass Loss: The MgF_2_ coating significantly reduced the biodegradation rate compared to uncoated referenced. While both alloys exhibited time-dependent corrosion, the Mg-Zn_R system displayed superior early-stage stability; its mass loss at day 3 (3.099%) was notably lower than its neodymium-based counterpart. Conversely, the Mg-Nd_R alloy showed a more rapid onset of degradation, losing 1.536% of its mass within the first 24 h—nearly triple the rate of the zinc-based sample (0.455%) during the same period. By day 14, both materials reached their maximum degradation levels of 6.855% (Mg-Zn) and 7.833% (Mg-Nd), respectively	Laboratory developed human patella-derived osteoblastic cell line Viability**:** Showed a 25% increase in cell proliferation at moderate extract concentrations (250 μg/mL) Apoptosis/Necrosis**:** Flow cytometry (Annexin V-FITC/PI) showed reduced necrosis—dropping from 8.6% (control) to 4.9% (Mg-Nd_I)—indicating that the controlled Mg^2+^ release and MgF_2_ surface are bio-stimulatory rather than toxic	Manescu (Paltanea) et al. [28]
Hydrothermal method	Mg-Al-Zn-Mn-Ca (AZ91-3Ca)	CaP layer identified as hydroxyapatite and related phosphate phases	Immersion tests Mass Loss**:** The CaP-coated Mg-based samples exhibited significantly lower degradation rates compared to the bare alloy. After 14 days of immersion, the mass loss for the coated samples was reduced to approximately 0.6 mg/cm^2^, whereas the uncoated AZ91-3Ca showed significantly higher mass loss. CR comprised between 1.73 ± 0.31 mm/year for uncoated samples and 0.72 ± 0.21 mm/year for coated ones Hydrogen Evolution**:** The coated samples displayed a much slower and more stable hydrogen evolution rate, confirming that the CaP layer acted as an effective physical barrier against the aggressive SBF environment.	MC3T3-E1 (mouse pre-osteoblast cells) The coated surfaces showed enhanced cell attachment and proliferation compared to the bare alloy. The CaP coating provided a more favorable surface chemistry for osteoblasts adhesion and metabolic activity	Ali et al. [32]
One-pot hydrothermal synthesis	Mg-Zn-Zr (ZK60)	Strontium (Sr)-doped HAp, structured as nanorods/nanowires	Electrochemical measurements and immersion tests Corrosion Resistance**:** The Sr-doped HAp coating significantly improved the corrosion resistance of the ZK60 alloy. The corrosion current density i_corr_ of the coated sample was about 5.09 × 10^−6^ A/cm^2^ (HAp-coated) and 2.357 × 10^−6^ A/cm^2^ (3Sr-HAp), a value that is two orders of magnitude lower than 1.46 × 10^−4^ A/cm^2^ (bare ZK60) Hydrogen Evolution**:** After 14 days of immersion, the hydrogen evolution volume for the Sr-doped HAp coated sample was significantly reduced compared to the uncoated substrate	BMSCs (bone marrow stem cells) The Sr-doped HAp nanorod/nanowire morphology exhibited excellent cytocompatibility. The presence of Sr was shown to significantly promote the proliferation and differentiation of BMSCs compared to pure HAp coatings	Wang et al. [33]

RE—rare earth elements; i_corr_—corrosion current density; accumulated CR—accumulated corrosion rate; 8-HQ—hydroxyquinoline; TCP—tricalcium phosphate; HF—hydrofluoric acid.

**Table 2:** Decision table regarding the main conversion coating technique, including coating type, processing parameters, corrosion investigation, bioactivity/biocompatibility, advantages, drawbacks, and decision indications.

Ref.	Coating Type	Composition	Key Processing Parameters	Corrosion Resistance	Bioactivity/Biocompatibility	Advantages	Limitations	Decision Indication
Rudd et al. [36], Dabala et al. [37], Han et al. [39], Cui et al. [40], Cui et al. [41]	Rare earth chemical conversion	Ce^2+^, Nd^2+^, Y^3+^ ions, carboxylates, phosphates	Temperature, immersion, pH-controlled solutions	Medium and high corrosion resistance for coated samples	Medium osteoconductivity	Simple and low-cost method	Films can be brittle and thin	Adequate for orthopedic implants with medium corrosion control
Prabhu et al. [65],Zaludin et al. [66],Dong et al. [68],Han et al. [69],Lv et al. [70],You et al. [71],Li et al. [72],Su et al. [73]	Phosphate conversion	HAp, TCP, DCPD	Phosphate/calcium solution, other post-treatments are necessary (hydrothermal)	Medium and high corrosion resistance for coated samples	High osteoconductivity	Biomimetic behavior, good cell adhesion, controllable chemical composition	Porosity could induce local corrosion processes	Suitable to be used for bone defect treatments
Li et al. [34],Penuela-Cruz et al. [42],White et al. [43],Halimovic et al. [44],Wang et al. [45],Li et al. [46],Han et al. [69],Lv et al. [70]	MAO/PEO with bioactive elements incorporation	MgO matrix + Ca-P, TiO_2_, ZrO_2_ or other dopants	High-voltage PEO, electrolyte composition control, porosity control, short time process	High dense or porous films with enhanced corrosion protection due to dopants	Good to excellent biocompatibility	Thick layer, increased adherence, tunable porosity	Expensive equipment. Thermal stresses could induce microcracks of the coating	Best for load bearing implants requiring strong coating
Lin et al. [47],Hamdy and Butt [48],Kumar et al. [49]	Stannate/Ni-P conversion underlayer	Na_2_SnO_3_, Ni-P	Room temperature immersion, electroless deposition	Overall good corrosion behavior with a high increase resistance when Ni-P overlay is used. Self-healing possibility	Moderate; can improve bioactivity based on further layer deposition	Self-healing property	Multi-step process, Ni-P film requires careful control, limited bioactivity	Use when corrosion protection is firstly searched and bioactivity is a secondary feature
Talha et al. [50],Rodriguez-Alonso et al. [51],Meng et al. [55],Liu et al. [56],Song et al. [57], Nezamdoust et al. [58],Huang et al. [59], Peng et al. [60],Liu et al. [61],Kunjukunju et al. [62]	Hybrid sol–gel/Polymer-sealed coatings	SiH_4_, ZnO, PDA, chitosan, LbL polymer assemblies	Dip-coating or electrochemical methods, curing, LbL assembly	Moderate corrosion resistance due to polymer sealing that reduces porosity	Moderate to high biocompatibility/bioactivity. Dopants or polymers can improve cell adhesion process	Bioactive properties, multifunctional corrosion protection	Layer thickness limited, uncertain long term stability under applied mechanical load	Adequate for surface functionalization, thin protective bioactive films
Manescu et al. [28], Quan et al. [52], Liu et al. [56]	Fluoride/MgF_2_/Polymers	MgF_2_ ± polymer (PDA)	Fluoride treatment, immersion or chemical reactions	Moderate to high corrosion resistance	Moderate; can improve osteoblast or endothelial cell attachment	Simple process, corrosion resistance	Brittle, thin layer that needs polymer sealing	Suitable for cardiovascular or orthopedic implants with moderate degradation rates

**Table 3:** Detailed analysis of process parameters with devoted attention to testing conditions of different studies presented in Table 2.

Study	Coating Type (Table 2)	Process Parameters	Testing Conditions	Critical Limitation/Critical Advantage
Rudd et al. [36]	Rare earth chemical conversion	Immersion in cerium salt solution (CeCl_2_–based); presence of oxidizing agents (H_2_O_2_); treatment time: minutes range; pH: mildly acidic to neutral	Electrochemical testing: NaCl-based electrolyte; room temperature; electrochemical polarization, EIS	Non-physiological electrolyte
Dabala et al. [37]		Ce(NO_3_)_3_ solution; use of oxidizers, immersion time: 10–60 min; pH: controlled in acidic range	Immersion medium: NaCl solution; room temperature (20–25°)	Corrosion was investigated in marine-like conditions
Han et al. [39]		Y-based pretreatment followed by phosphating; solution contains Y-based salts and phosphate species, immersion time (10–30 min); ambiental temperature	NaCl solution; EIS and potentiodynamic polarization analysis; 25 °C	No physiological simulation; lacks temperature control at 37 °C
Cui et al. [40]		Nd salt and organic acid; immersion-based conversion process; 10–60 min; pH: mildly acidic	NaCl solution; polarization and EIS; room temperature	Organic-modified system but non-biological type medium
Cui et al. [41]		Nd salt immersion; post-treatments; time-dependent growth mechanism investigated	NaCl solution; EIS and polarization; room temperature	No standardized physiological testing. Post-treatment effects not evaluated under in vivo-like conditions
Prabhu et al. [65]	Phosphate conversion	Immersion in SBF; static immersion process; time: hours to days; temperature: 37 °C; pH: 7.4	SBF (Kokubo-type); 37 °C; immersion tests and electrochemical analysis (EIS/polarization)	High biomedical relevance
Zaludin et al. [66]		Immersion in Ca^2+^/PO_4_^3−^ aqueous solution; room temperature; time: tens of minutes to hours	NaCl solution (3.5 wt%), and/or SBF (limited use); room temperature; polarization analysis and EIS	Mixed testing environments; partial physiological relevance
Dong et al. [68]		Initial phosphate conversion layer; hydrothermal treatment at 120–180 °C; time: several hours; autoclave conditions	NaCl or SBF; room temperature (electrochemical testing), 37 °C (immersion); EIS, polarization, adhesion tests	Advanced processing testing is still partially non-standardized
Han et al. [69]		Step 1: PEO; step 2: immersion in Ca-P solution; temperature: 37 °C for Ca-P deposition; time: hours to days	SBF; temperature: 37 °C; immersion and electrochemical tests	Good physiological simulation; hybrid coating strategy
Lv et al. [70]		Electrolyte: silicate/phosphate-based MAO solution; voltage/current-controlled regime; treatment time: minutes; temperature: near ambient	NaCl or SBF; room temperature; polarization, EIS, immersion	Strong coating control; limited physiological temperature usage
You et al. [71]		Multi-step Ca-P deposition; morphology tuning; temperature: 37 °C	SBF; 37 °C; corrosion analysis and cell compatibility tests	Strong alignment with biomedical application
Li et al. [72]		Chemical conversion and composite deposition; presence organic (phytic acid) and bioactive glass particles; room temperature; time: controlled immersion	SBF; 37 °C; immersion degradation and electrochemical analysis	Biofunctional coating with good physiological relevance
Su et al. [73]		Step 1: Ca-P conversion coating; step 2: fluoride treatment (NaF solution); room temperature; time: sequential immersion steps	SBF; 37 °C; immersion degradation and electrochemical analysis	Improved corrosion resistance; physiologically relevant testing
Li et al. [34]	MAO/PEO with bioactive elements incorporation	Electrolyte: silicate-based and yttrium nitrate additive, voltage-controlled MAO regime; treatment time: minutes (maximum 20 min.); room temperature	NaCl solution; room temperature: EIS and polarization analysis	Parameter optimization study; non-physiological testing
Penuela-Cruz et al. [42]		Electrolyte enriched with Ti species; PEO under controlled voltage/current; treatment time: minutes	NaCl solution; room temperature; electrochemical analysis and immersion tests	Composite oxide design; lacks physiological testing
White et al. [43]		Electrolyte containing Ti precursors; high-voltage plasma discharge regime; treatment time: minutes	NaCl solution; room temperature; polarization and EIS	Early stage TiO_2_ functionalization; engineering corrosion focus
Halimovic et al. [44]		Step 1: PEO in Ti-containing electrolyte; step 2: polymer sealing (PLA); time: minutes (PEO and post-treatment)	NaCl and SBF; room temperature; corrosion analysis and barrier evaluation	Hybrid (ceramic + polymer); partial biomedical orientation
Wang et al. [45]		Electrolyte with Zr-containing species; voltage/current optimization; treatment time: minutes	NaCl solution; room temperature; EIS, polarization, and phase analysis	Focus on phase transformation; non-physiological testing
Li et al. [46]		Electrolyte engineered for pore size modulation; MAO/PEO voltage control; treatment time: minutes	NaCl solution; room temperature; electrochemical analysis and morphology correlation	Structure–property focus; limited biomedical testing
Lv et al. [70]		Electrolyte: silicate/phosphate system; voltage-controlled MAO; treatment time: minutes	NaCl and SBF; room temperature; EIS and polarization analysis	Biomedical alloy but testing not fully standardized
Lin et al. [47]	Stannate/Ni-P conversion underlayer	Immersion in stannate solution (Na_2_SnO_3_); alkaline pH; time: minutes to tens of minutes; elevated temperature (60–80 °C)	NaCl solution; room temperature; polarization method	Classical conversion system; non-biological testing
Hamdy and Butt [48]		Stannate bath with inhibitors; immersion-based coating formation; time: tens of minutes	NaCl solution; room temperature; electrochemical analysis and self-healing evaluation	Functional coating; still marine-type testing
Kumar et al. [49]		Stannate conversion layer; electroless Ni-P deposition; temperature: controlled plating conditions (80–90 °C for Ni-P)	NaCl solution; room temperature; corrosion and tribo-mechanical analysis	Multi-layer engineering system; not biomedical-oriented
Talha et al. [50]	Hybrid sol–gel/Polymer-sealed coatings	Silane sol preparation and ZnO nanoparticle incorporation; dip-coating/self-assembly; curing step (moderate temperature 80–120 °C)	NaCl solution; room temperature; EIS and polarization analysis	Hybrid barrier coating; non-physiological testing
Rodriguez-Alonso et al. [51]		Sol–gel synthesis (siloxane network); doping with corrosion inhibitors; dip-coating and curing (100–150 °C)	NaCl solution; room temperature; electrochemical testing and immersion analysis	Environmentally friendly inhibitors; no physiological simulation
Meng et al. [55]		Dopamine self-polymerization (alkaline solution, pH of 8.5); Zn^2+^ incorporation post-deposition; temperature: ambient; time: hours	SBF or PBS; 37 °C; immersion and electrochemical analysis	Strong biofunctional orientation
Liu et al. [56]		Step 1: fluoride conversion treatment (HF); step 2: PDA deposition; room temperature	SB/physiological buffer; 37 °C; immersion test and electrochemical characterization	Clinically oriented (vascular stents)
Song et al. [57]		Conversion coating and embedded inhibitor-loaded microcapsule; immersion; room temperature	NaCl solution; room temperature; electrochemical analysis and self-healing evaluation	Smart coating; not physiologically tested
Nezamdoust et al. [58]		Sol–gel synthesis (organic–inorganic hybrid); dip-coating and thermal curing; temperature: 100–150 °C	NaCl solution; room temperature; corrosion analysis and mechanical evaluation	Engineering coating; lacks biomedical testing
Huang et al. [59]		In situ electropolymerization, controlled in potential/current; electrolyte: pyrrole-containing solution; room temperature	NaCl solution; room temperature; EIS and polarization analyses	Conductive polymer coating; non-physiological
Peng et al. [60]		One-step electrochemical deposition; cyclic voltammetry control; silane coupling and PPy growth	NaCl solution; room temperature; electrochemical tests	Hybrid electrochemical coating; engineering focus
Liu et al. [61]		Alternating adsorption of polyelectrolytes; controlled pH and ionic strength; room temperature	SBF or PBS; 37 °C; immersion and electrochemical test	Good physiological relevance
Kunjukunju et al. [62]		LbL assembly of bioactive polymers; surface pre-treatment required; room temperature	SBF/cell culture media; 37 °C; corrosion and bioactivity tests	Strong biomedical relevance (biofunctional coatings)
Manescu et al. [28]	Fluoride/MgF_2_/Polymers	No dedicated chemical conversion coating protocol as primary variable; immersion in HF as a manufacturing step consequence	SBF/cell culture media; 37 °C; immersion degradation tests, biofunctional assays (cell viability, osteogenic response)	High clinical relevance; emphasizes biological response
Quan et al. [52]		Immersion in HF; formation of MgF_2_ conversion coating; time: seconds to minutes; room temperature	SBF; 37 °C; immersion and electrochemical analysis	Good standardization toward biomedical conditions
Liu et al. [56]		Step 1: HF-treatment (MgF_2_ coating); step 2: polydopamine deposition via dopamine self-polymerization; pH 8.5; time: hours	SBF; 37 °C; electrochemical tests, biocompatibility assays	Hybrid biofunctional coating with strong translational relevance

## Data Availability

The original contributions presented in the study are included in the article; further inquiries can be directed to the corresponding authors.
